# Increased Glucose Availability Sensitizes Pancreatic Cancer to Macrophage-Targeting Immunotherapies

**DOI:** 10.1158/2767-9764.CRC-25-0338

**Published:** 2026-06-23

**Authors:** Jonathan J. Hue, Mehrdad Zarei, Priyashree Sunita, Sami O. Abul-Khoudoud, Goutam Dey, Hallie J. Graor, Katie E. Blise, Dove Keith, Shamilene Sivagnanam, Shakti P. Pattanayak, Erryk S. Katayama, Omid Hajihassani, Charles D. Lopez, Rosalie C. Sears, Robert Eil, Lisa M. Coussens, Jonathan R. Brody, Ali Vaziri-Gohar, Jordan M. Winter

**Affiliations:** 1 Division of Surgical Oncology, Department of Surgery, University Hospitals Cleveland Medical Center, Cleveland, Ohio.; 2 Department of Surgical Oncology, University of Texas MD Anderson Cancer Center, Houston, Texas.; 3 Case Comprehensive Cancer Center, Case Western Reserve University, Cleveland, Ohio.; 4 Brenden-Colson Center for Pancreatic Care, Oregon Health & Science University, Portland, Oregon.; 5 Department of Cell, Developmental and Cancer Biology, Knight Cancer Institute, Brenden Colson Center for Pancreatic Care, Oregon Health & Science University, Portland, Oregon.; 6 Department of Biochemistry, School of Medicine, Case Western Reserve University, Cleveland, Ohio.; 7 Case Western Reserve University, Cleveland, Ohio.; 8 The Ohio State University College of Medicine, Columbus, Ohio.; 9 Knight Cancer Institute, Oregon Health & Science University, Portland, Oregon.; 10 Department of Cell, Development and Cancer Biology, Oregon Health & Science University, Portland, Oregon.; 11 Department of Molecular and Medical Genetics, Oregon Health & Science University, Portland, Oregon.; 12 Division of Surgical Oncology, Department of Surgery, Knight Cancer Institute, Oregon Health & Science University, Portland, Oregon.; 13 Department of Cancer Biology, Stritch School of Medicine, Loyola University Chicago, Maywood, Illinois.; 14 Department of Surgery, Stritch School of Medicine, Loyola University Chicago, Maywood, Illinois.

## Abstract

Pancreatic ductal adenocarcinoma (PDAC) is characterized by an immunosuppressive tumor microenvironment (TME) that limits the efficacy of immunotherapies. Many tumor-suppressive immune cells, including inflammatory macrophage subsets, rely on glycolysis to sustain effector function; however, the pancreatic TME is relatively glucose-limited. In this study, we investigated whether increasing glucose availability in the periphery, which in turn translates to increased intratumoral glucose, could enhance immune-based therapies in PDAC. *In vitro*, glucose restriction induced metabolic reprogramming of inflammatory (M1-like) macrophages toward an oxidative, M2-like state with reduced inflammatory effector markers. In immunocompetent mice, administration of 30% dextrose drinking water increased both peripheral and intratumoral glucose levels and modestly shifted tumor transcriptional profiles toward a more inflammatory state without altering overall immune cell abundance. When macrophage-targeting immunotherapies (colony stimulating factor 1 receptor inhibition with PLX3397 or C–C chemokine receptor type 2 inhibition with PF-4136309) were combined with systemic hyperglycemia, tumors exhibited a pronounced increase in iNOS^+^ M1-like macrophages, a reduction in arginase^+^ M2-like macrophages, enhanced CD8^+^ T-cell infiltration, and decreased abundance of tumor-associated fibroblasts. These immunotherapies improved overall survival in immunocompetent mice bearing orthotopic pancreatic tumors only when combined with hyperglycemia. Analyses of patient samples confirmed the presence of a favorable antitumor immune infiltrate with elevated glucose levels. Together, these findings identify glucose availability as a key regulator of macrophage polarization and immunotherapy efficacy in PDAC.

**Significance:**

PDAC is largely resistant to immunotherapies. Our findings identify glucose availability in the TME as a modifiable determinant of macrophage polarization and immunotherapy response. In preclinical models, transient hyperglycemia was associated with enhanced efficacy of macrophage-targeting immunotherapies and promoted a more inflammatory TME.

## Introduction

Pancreatic cancer remains a devastating disease. The 5-year overall survival rate for patients with metastases has increased by only 1% over the last 15 years: from 2% in 2012 to 3% in 2026 (1, 2). This limited progress reflects the fact that pancreatic ductal adenocarcinoma (PDAC) is largely resistant to novel and conventional systemic therapies (3–5). A defining feature of PDAC is the dense, metabolically hostile, and immunosuppressive tumor microenvironment (TME). A tenacious stromal reaction also results in restricted nutrient availability, exemplified by low intratumoral glucose levels (6). Together, these features are thought to restrict immune cell infiltration and function in PDAC, contributing to immunotherapy resistance.

Tumor-associated macrophages represent a dominant immune population within the PDAC TME (7). Macrophages exist on a spectrum with diverse roles and are broadly classified as inflammatory and tumor-suppressive (M1-like) or immunosuppressive and tumor-promoting (M2-like; ref. 8). M2-like macrophages predominate in many PDAC tumors and are associated with tumor growth, fibrosis, and suppression of cytotoxic T-cell activity. Consistent with this framework, the total number of macrophages and the intratumoral M1:M2 ratio carry prognostic significance (9–12). Thus, strategies that favorably affect macrophage polarization have been explored as potential therapies, yielding novel macrophage-targeting treatments. For instance, colony stimulating factor 1 receptor (CSF1R) inhibitors and C–C chemokine receptor type 2 (CCR2) inhibitors have been used in clinical trials including patients with PDAC (13–16). Although CSF1R inhibitors have received FDA approval for certain rare tumors, neither class has demonstrated clinical efficacy in patients with PDAC.

Metabolic conditions are recognized as important determinants of immune cell phenotype and function. Inflammatory immune cells, including M1-like macrophages, primarily rely on glycolysis for energy production and effector functions such as nitric oxide synthesis and cytokine production (8). Glycolysis is an inefficient ATP-generating process, so these immune cells require a steady supply of glucose substrate to produce sufficient ATP. As glucose is relatively scarce in the PDAC TME relative to normal pancreatic tissue (6), these antitumor immune cells may be at a disadvantage, contributing to the “cold” immunophenotype of PDAC. Conversely, tumor-promoting M2-like macrophages preferentially favor oxidative phosphorylation, which is a much more efficient means of energy production than glycolysis alone, enabling these immune subsets to function well when glucose levels are limited in the PDAC TME (7, 8).

We hypothesize that these intrinsic metabolic differences between immune cell subsets can be exploited to enhance immunotherapy efficacy. Strategies that modify the TME to render existing immunotherapies more potent against PDAC are compelling given the persistent lack of effective systemic therapies. Intentional manipulation of intratumoral glucose levels represents an example of this approach. Herein, we perform *in vitro* metabolic analyses and *in vivo* experiments with dietary glucose manipulations to determine the impact on macrophage-targeting immunotherapy effectiveness. Our findings reveal that glucose availability is required to sustain inflammatory macrophage metabolism, intentional hyperglycemia alters the immune landscape of pancreatic tumors favorably, and elevating intratumoral glucose levels enhances the efficacy of macrophage-modulating immunotherapies in immunocompetent settings.

## Materials and Methods

### Cell lines and reagents

For *in vitro* macrophage experiments, bone marrow was harvested from bilateral femurs and tibias of C57Bl/6J mice at approximately 10 to 12 weeks of age. Marrow was initially cultured on a Petri dish in complete Dulbecco’s Modified Eagle Media (DMEM) supplemented with 15% macrophage colony-stimulating factor (M-CSF; L929 cell culture supernatant; ref. 17). On day three of culture, an additional 2 mL of DMEM with 15% M-CSF was added to each plate. After 7 days of culture, assays involving bone marrow–derived macrophages (BMDM) were initiated. Macrophages were polarized toward an inflammatory, M1-like phenotype using lipopolysaccharide (LPS; 20 ng/mL; Sigma-Aldrich; 297-473-0) and interferon gamma (IFNγ; 50 ng/mL; PeproTech; 315-05). Macrophages were polarized toward an immunosuppressive, M2-like phenotype using interleukin 4 (IL4; 20 ng/mL; PeproTech; AF-214-14). Throughout this report, M1-like macrophages are depicted in red, whereas M2-like macrophages are shown in blue. L929 cells were generously provided by the laboratory of Dr. Stanley Huang.

Murine pancreatic cancer cells (KPC K8484: KrasG12D; Trp53R172H/+; Pdx1-Cre) were provided by the laboratories of Drs. Darren Carpizo and Eric Collison. These KPC cells have been used extensively in prior publications from our group (6, 18). Formal cell line authentication was not performed for this study. Cells were routinely monitored for morphology and growth characteristics and were confirmed to be free of *Mycoplasma* contamination prior to use (MycoAlert Detection Kit, ATCC, 30-1012K). KPC cells utilized for experiments exhibited stable expression of luciferase. Cell lines were maintained at 37°C with 5% CO_2_. High-glucose experiments (25 mmol/L) utilized DMEM (Thermo Fisher Scientific, 11965092). Low-glucose experiments utilized no glucose DMEM (Thermo Fisher Scientific, 11966025) adjusted to specified glucose concentrations (1 or 2.5 mmol/L), as indicated and previously described (6). All culture media was supplemented with 10% fetal bovine serum (FBS), 1% penicillin/streptomycin, and prophylactic doses of Plasmocin (Life Technologies, MPP-01-03) to prevent *Mycoplasma* infection.

### qPCR analysis

RNA was extracted after 24 hours of designated treatment using a PureLink RNA isolation kit (Life Technologies, 12183025) and treated with DNase (Life Technologies, AM2222). cDNA was synthesized using 1 μg of total RNA (Thermo Fisher Scientific, 01127021). Primers for iNOS (Thermo Fisher Scientific, Mm00440502_m1) and CD206 (Thermo Fisher Scientific, Mm01329359_m1) as markers of M1-like and M2-like macrophages, respectively, were used and normalized to β-actin. All PCR reactions were performed in triplicate using iTaq Universal Probes Supermix (Bio-Rad, 1725131). RT-qPCR acquisition was performed using Bio-Rad CFX96.

### Western blot analysis

Total protein was extracted with RIPA buffer (Thermo Fisher Scientific, 89901) after 48 hours of specific cell culture conditions unless otherwise detailed. Lysates were supplemented with a protease–phosphatase inhibitor (Thermo Fisher Scientific, WC320075). Protein was quantified using the BCA Protein Assay (Thermo Fisher Scientific, 23225). Proteins were separated on 4% to 12% Bis-Tris Plus gels (Thermo Fisher Scientific, NM04120) and transferred to polyvinylidene difluoride membranes (Thermo Fisher Scientific, 2PM22091-01). Membranes were blocked for 1 hour in a 5% milk solution at room temperature and then probed with antibodies against iNOS [NOS2 (C-11); 1:1,500; Santa Cruz Biotechnology; sc-7271, RRID: AB_627810], arginase [arginase I (E-2); 1:5,000; Santa Cruz Biotechnology; sc-271430, RRID: AB_10648473], CSF1R (1:1,000; Proteintech; 25949-1-AP, RRID: AB_2880306), or CCR2 (1:1,000; Proteintech; 16153-1-AP, RRID: AB_2262945) overnight at 4°C. α-Tubulin (Cell Signaling Technology, 3873, RRID: AB_1904178) and β-actin (Proteintech; HRP-60008, RRID: AB_2819183) were used as loading controls, and normalized protein levels were calculated. Blots were probed with secondary antibodies customized for an Odyssey imaging system (LI-COR, D10831-15 or D10901-15).

### DCFDA assay

BMDMs were plated at approximately 80% confluency in 96-well plates. After 24 hours, media were changed (25 mmol/L vs. 2.5 mmol/L glucose) and macrophages were polarized toward an M1- or M2-like phenotype or left as naïve (M0) macrophages. After 48 hours, cells were washed with PBS, and then FBS-free media (25 mmol/L or 2.5 mmol/L glucose) was added to cells. Plates were incubated at 37°C with 5% CO_2_ for 1 hour. Media were aspirated and replaced with FBS-free media (25 or 2.5 mmol/L glucose) including 50 μmol/L H_2_-DCFDA (Invitrogen, D399) and incubated for another 30 minutes. Wells were washed and then analyzed using 475 nm fluorescence excitation and a fluorescence emission of 500 to 550 nm using a microplate reader (GloMax Explorer system, Promega).

### Cell viability and coculture assays

BMDM and KPC viability were assessed by double-stranded DNA quantitation (PicoGreen dsDNA assay, Life Technologies, P7589). Experiments were performed in both low- and high-glucose conditions. Increasing concentrations of immunotherapies (PLX3397 and PF-4136309) were also utilized in select experiments. All cell viability assays were performed after approximately 4 days of treatment based on maximal cell confluence.

For coculture experiments, approximately 10^5^ naïve BMDMs were plated in low- or high-glucose conditions and left to equilibrate 24 hours. After 24 hours, 10^4^ KPC cells were added to the plates, as well as IFNγ and LPS (for M1-like macrophages) or IL4 (for M2-like macrophages). KPC cell growth without added BMDMs was used as a negative control. Coculture experiments ended after 4 days. Given that the KPC cells utilized exhibit stable luciferase expression, KPC cell growth was measured by the addition of D-luciferin to culture media followed by luminescence quantification with a Promega GloMax Explorer. Baseline luminescence of BMDM was subtracted from measurements. Upon completion of the coculture experiments, colonies were washed and fixed in a crystal violet solution (BD Biosciences, 0296874). Photographs were obtained to visually depict cell viability.

### Metabolism assays

#### Seahorse extracellular flux analysis

Approximately 40,000 BMDM were seeded in cell culture plates. After 24 hours, culture media was changed (i.e., low- or high-glucose media), and IL4 or LPS and IFNγ were added. Oxygen consumption rate (OCR) and extracellular acidification rate (ECAR) were quantified using an Agilent Seahorse XFp analyzer (RRID: SCR_019545). Oligomycin (final concentration: 1.5 μmol/L) and rotenone/antimycin A (final concentration: 0.5 μmol/L) were provided in a Cell Mito Stress Test kit. Carbonyl cyanide 4-(trifluoromethoxy)phenylhydrazone (FCCP, final concentration: 50 μmol/L) was obtained from Sigma-Aldrich (370-86-5), and the optimal concentration was determined through dose–response assays. Assay data were normalized to cell number.

#### Liquid chromatography/mass spectrometry metabolomics

Naïve BMDMs were plated in 30-mm dishes at approximately 90% confluency and left for 24 hours. Media were then changed to high-glucose (25 mmol/L glucose) or low-glucose (1 mmol/L glucose) media, and IL4 or LPS and IFNγ were added. Cells were then left for 48 hours. Conditions were performed in triplicate. One additional plate was used to quantify protein concentration and confirm expected macrophage polarization via Western blot. After 48 hours, culture media were removed, and cells were rinsed with ice-cold PBS. Ice-cold 80% methanol was added to plates, and cells were scraped to extract intracellular metabolites. Cell lysates were centrifuged at 14,000 *g* at 4°C for 10 minutes. The supernatant was dried using a SpeedVac and then frozen at −80°C. Samples were shipped on dry ice, and assays were performed by the Mass Spectrometry Core at Beth Israel Deaconess Medical Center. Metabolites were quantified by liquid chromatography/mass spectrometry (LC/MS) analysis using 5500 QTRAP hybrid quadrupole mass spectrometer (AB-SCIEX) coupled to a Prominence High-Performance Liquid Chromatography system (Shimadzu Corporation) and amide HILIC chromatography (Waters) at pH 9.2 (19). Metabolite fractions from each sample were normalized to total protein concentration.

### Glucose measurements

#### In vitro

The glucose concentration in cell culture media was quantified using GlucCell glucose test strips (CESCO Bioengineering Co., Ltd., GC001001), as previously described (20). Measurements were performed in triplicate.

#### *In vivo*, serum

Mice were briefly anesthetized using isoflurane gas. A 1-mm section of their tail was sharply removed using a scalpel. The initial drop of blood was wiped clean. Glucose concentration in the subsequent drop of blood was quantified using a glucometer (Abbot). Measurements were taken 2 to 3 weeks after initiation of 30% dextrose water (D30) treatment.

#### *In vivo*, intratumoral

Tumors of mice at a survival endpoint were removed via perimortem laparotomy. Tumors were washed once in ice-cold PBS, cut into 2-mm pieces, and subsequently homogenized. Glucose was quantified using a Promega Glucose-Glo assay (J6021).

### Flow cytometry

Mice with confirmed tumors were first randomized to 7 days of D30 or standard water *ad libitum*. Mice were then randomized again to receive vehicle or an immunotherapy for an additional 10 days while continuing their assigned water. Single-cell suspensions were prepared from freshly harvested tumor tissue. Solid tumors were mechanically dissociated and enzymatically digested using collagenase IV (1 mg/mL) and DNase I (50 U/mL) at 37°C for 30 to 45 minutes, followed by filtration through a 70-μm strainer. Red blood cells were lysed using ACK lysis buffer for 2 to 3 minutes at room temperature. Cells were washed twice with PBS containing 2% FBS and counted using trypan blue exclusion.

For surface marker analysis, 1 to 2 × 10^6^ cells were resuspended in 100 μL FACS buffer (PBS, 2% FBS, and 1 mmol/L EDTA). To prevent nonspecific binding, cells were incubated with Fc receptor–blocking reagent for 10 minutes at 4°C. Cells were then stained with fluorochrome-conjugated monoclonal antibodies against immune cell surface markers for 30 minutes at 4°C in the dark. Following staining, cells were washed twice with FACS buffer. After surface staining, cells were fixed and permeabilized (BD Cytofix/Cytoperm Fixation/Permeabilization Kit; 554714) according to the manufacturer’s instructions. Intracellular staining was performed for 30 to 45 minutes at room temperature in the dark, followed by washing and resuspension in FACS buffer. Antibodies used included CD45-PerCP-Cy5.5 (BioLegend cat. #110728, RRID: AB_893346), CD3e-PE (Thermo Fisher Scientific cat. #12-0031-82, RRID: AB_465496), CD4-APC (BioLegend cat. #100411, RRID: AB_312696), CD8-FITC (BioLegend cat. #140403, RRID: AB_10641694), F4-80-Pacific Blue (Thermo Fisher Scientific cat. #MF48028, RRID: AB_10373419), iNOS-Alexa Fluor 488 (Thermo Fisher Scientific cat. #53-5920-82, RRID: AB_2574423), Arginase-APC (Thermo Fisher Scientific cat. #17-3697-82, RRID: AB_2734835), programmed cell death-ligand 1 (PD-L1)-Alexa Fluor 647 (Cell Signaling Technology cat. #50625), CD140a (PDGFRα)-PE (BioLegend cat. #135905, RRID: AB_1953268), B220-PerCP (BioLegend cat. #103205, RRID: AB_312990), and Nk1.1-PE (BioLegend cat. #137607, RRID: AB_10612749).

Total events were first gated based on forward- and side-scatter (FSC-A vs. SSC-A) to exclude debris, followed by singlet discrimination using FSC-A versus FSC-H. Viable leukocytes were identified and gated based on FSC-A and SSC-A properties, after which CD3^+^ T cells were selected and further subdivided into CD4^+^ and CD8^+^ T-cell populations. Macrophages were gated on F4/80^+^ and were further characterized based on expression of iNOS and arginase-1 to define inflammatory (iNOS^+^, M1-like) and immunosuppressive (arginase-1^+^, M2-like) phenotypes. Additional lineage markers, including NK1.1, B220 (B cells), CD140α (fibroblasts), and PD-L1, were used to delineate specific immune subpopulations as indicated. Percentages shown in each gate represent the proportion of parent populations. All gates were set based on fluorescence-minus-one controls and applied uniformly across all samples. Analyses were performed using FlowJo 10.8.1 (RRID: SCR_008520).

### Bulk RNA sequencing

C57Bl/6J mice bearing orthotopic pancreatic tumors were given standard drinking water (*n* = 5) or D30 water (*n* = 5) *ad libitum* for a total of 4 weeks after tumor implantation. Tumors were harvested, immediately placed in ice-cold PBS, and then flash frozen. Samples were processed by Novogene. Raw gene counts were normalized to control samples, and differential expression analysis was performed. For focused analyses, expression of immune-, macrophage-, and tumor-associated transcripts were examined, including inflammatory and immunosuppressive markers (IFNγ, TNFα, IL1β, IL6, CD69, FOXP3, IL10, CTLA-4, TGFβ, and PD-1) as well as common immune lineage and macrophage genes (CD45, CD11b, F4/80, iNOS, CD80, CD86, CD206, CD301, CD163, arginase, CSF1R, and CCR2).

In a separate experiment, unbiased Gene Ontology (GO) enrichment analyses were conducted to identify pathways altered across treatment conditions. Mice with established orthotopic pancreatic tumors were randomized to receive D30 water or standard drinking water for 7 days. Mice subsequently underwent a second randomization to receive PLX3397 or vehicle for an additional 7 days while continuing their assigned water regimen. Tumors were harvested, washed once in ice-cold PBS, placed in RNAlater, snap-frozen in liquid nitrogen, and stored at −80°C. Samples were shipped on dry ice to Azenta Life Sciences for RNA extraction, library preparation, and bulk RNA sequencing (RNA-seq). Sequencing quality control, read alignment, differential gene expression analysis, and GO enrichment analysis were performed by Azenta Life Sciences using standard, validated bioinformatics pipelines.

### Immunohistochemistry staining

Orthotopic pancreatic tumors from immunocompetent mice were fixed in formalin following resection. Tumors were sectioned and stained for Ki-67, cleaved caspase-3, iNOS, and CD163.

### Tumor injections in mice

All experiments involving mice were approved by the Case Western Reserve University Institutional Animal Care Regulations and Use Committee (protocol 2018-0063). Mice were maintained under pathogen-free conditions in a dedicated animal facility. Eight-to-ten-week-old, C57Bl/6J (RRID: IMSR_JAX:000664) and athymic nude (RRID: IMSR_JAX:002019) mice were purchased from The Jackson Laboratory. NOD/SCID gamma (NSG, RRID: IMSR_JAX:005557) mice were graciously provided by the laboratory of Dr. Andrew Sloan.

For subcutaneous flank injections, 1 × 10^6^ cells were suspended in 200 μL of a 1:1 PBS:Matrigel solution and injected into the subcutaneous tissue overlying the right flank. Treatment was initiated once tumors were palpable (120–150 mm^3^). Experiments were terminated once clinically significant ulceration was noted or mice showed signs of distress.

For orthotopic injections, mice were anesthetized using inhaled isoflurane gas and were shaved, prepped, and draped in a sterile fashion. After achieving an appropriate depth of anesthesia, a 5 mm left subcostal incision was made, and the peritoneal cavity was entered. The spleen and tail of the pancreas were externalized. For orthotopic pancreatic tumors, 30,000 KPC cells were suspended in 30 μL of a 40:60 mixture of Matrigel (Corning, 354234) and PBS. The suspension was carefully injected into the pancreatic tail. The pancreas was not manipulated for 60 seconds to limit leakage. The pancreas and spleen were returned to the peritoneal cavity, and the incision was closed with a permanent suture followed by a skin clip. For hepatic metastases models, 100,000 KPC cells were suspended in 200 μL of PBS, and the suspension was carefully injected into the splenic parenchyma, as previously described (21). Direct pressure was held at the puncture site for 2 to 3 minutes to ensure hemostasis. The spleen was not manipulated for a total of 5 minutes after injection to allow for adequate cell transit into the splenic vasculature. The splenic hilum was ligated with a 4-0 silk suture, and the spleen was removed sharply to ensure any morbidity of the procedure was related to metastatic spread and not the tumor injection site. The pancreas was returned to the peritoneal cavity, and the incision was closed with a permanent suture followed by a skin clip. Mice were removed from the anesthetic gas and monitored during recovery. On postoperative day 10, the presence of viable pancreatic or liver tumors was confirmed with bioluminescence imaging using 100 μL intraperitoneal injection of D-Luciferin (50 mg/mL, PerkinElmer, 2898979). Mice with confirmed tumors were then randomized to the indicated treatment groups. Mice without a bioluminescence signal were not used in experiments. Treatment was uniformly initiated on postoperative day 14 for survival experiments.

Mice randomized to high-glucose groups were provided D30 water *ad libitum*, as previously described (6). Treatments included vehicle (10% PEG-400, 4% Tween-80, and 86% saline), pexidartinib/PLX3397 (50 mg/kg, daily, oral gavage; MedChemExpress, HY-16749), or PF-4136309 (10 mg/kg, twice daily, oral gavage, MedChemExpress, HY-13245; refs. 16, 22, 23).

### Multiplex immunohistochemistry analysis of human metastatic PDAC samples

Multiplex immunohistochemistry (mIHC; refs. 24, 25) was performed on biopsy samples from an independent Oregon Health & Science University (OHSU) cohort of patients with PDAC liver metastases who had received systemic therapy. This experiment was approved by the Institutional Review Board at OHSU (Protocol 19211), and all patients provided written informed consent. All procedures were conducted in accordance with the ethical principles outlined in the Declaration of Helsinki. In total, biopsies from 11 patients were collected, and 68 regions of interest (ROI) were analyzed, including 49 ROIs from hyperglycemic patients (*n* = 8) and 19 ROIs from nonhyperglycemic patients (*n* = 3). Hyperglycemia was defined as a peripheral glucose measurement of more than 180 mg/dL preceding the index biopsy. mIHC staining and image processing were performed following a previously published protocol (24, 26). Briefly, images were acquired at 20× on Leica AT2 scanners. Images were registered using the SURF algorithm in MATLAB. Signal deconvolution was performed in FIJI, single cells were segmented using StarDist (27), and single-cell measurements were taken using CellProfiler (28). Cell phenotyping was conducted using a gating strategy to delineate 11 immune lineages, and cell densities were quantified for each ROI. ROI-level immune cell densities were grouped by patient glycemic status (hyperglycemic vs. nonhyperglycemic) and summarized at the group level to compare immune composition between conditions. Functional markers (Ki-67, PD-L1, and HLA-II) were used to further analyze myeloid cells. Spatial proximity analyses were also performed following previously published methods (29). Briefly, the number of times cells of two phenotypes were located within 20 μm from one another in the tissue was calculated. The frequency of these proximities was then normalized by the densities of the cells involved in the proximity and log_10_ transformed.

### Statistical analyses

All *in vitro* experiments were performed in duplicate at a minimum with the exception of selected supplemental Western blots. For qPCR experiments, cell viability assays, and macrophage functional assays, data are presented as the mean ± SEM. Quantification used for Western blot analyses were normalized to the specified loading control. All data points are presented for intratumoral glucose concentrations, peripheral glucose concentrations, and body weights. The Wilcoxon rank-sum test was used to make statistical comparisons of continuous data between treatment groups given the small sample sizes and potential for nonnormal data distributions. Median survival was assessed for orthotopic experiments using the Kaplan–Meier method and was compared using the log-rank test. Statistical comparisons were made using StataSE v16.1 (StataCorp LLC, RRID: SCR_012763), and graphical displays were made using GraphPad Prism version 9 (GraphPad Software, RRID: SCR_002798). A *P* value was considered statistically significant when <0.05 (*, *P* < 0.05; **, *P* < 0.01; ***, *P* < 0.001; ****, *P* < 0.0001).

## Results

### Immunocompetence is required to suppress PDAC growth *in vivo*

To establish the importance of a complete immune repertoire in controlling PDAC progression, KPC cells were injected into the pancreas of mice with varying degrees of immunocompetence (Supplementary Fig. S1A). Untreated mice with incomplete immune systems, including both NSG (deficient in T, B, and NK cells with impaired innate immune function; ref. 30) and athymic nude mice (T-cell dysfunction; ref. 30), developed spontaneous liver metastases and rapidly died (Supplementary Fig. S1B). Untreated immunocompetent C57Bl/6J mice survived more than twice as long and did not develop evidence of metastatic spread (Supplementary Fig. S1C). These data indicate that an intact immune system plays a major role in limiting PC growth, metastatic potential, and survival in conventional preclinical models and support a biologic rationale for targeting immune components in the PDAC TME.

### Macrophage polarization differentially regulates cancer cell growth and is dependent on glucose availability *in vitro*

To demonstrate the distinct function of macrophage subsets *in vitro*, M1-like and M2-like macrophages were cocultured with KPC cells. Relative to KPC cells alone, the addition of M1-like macrophages resulted in a 95% reduction in KPC viability. M2-like macrophages increased KPC growth by approximately 40% (Supplementary Fig. S2). This experiment was performed in supraphysiologic glucose conditions (25 mmol/L glucose), which should promote optimal M1-like macrophage function based on prior insights of macrophage metabolism (8). The same experiment was then repeated under low-glucose conditions to better mimic the pancreatic TME. M1-like macrophages were relatively less effective at inhibiting KPC growth, whereas M2-like macrophages continued to promote KPC growth (Supplementary Fig. S3A). Macrophage stimulants alone did not dramatically affect KPC survival in high- or low-glucose conditions. KPC cells exhibited reduced growth in low-glucose conditions (Supplementary Fig. S3A and S3B).

After 5 days in culture, glucose concentrations in M1-like macrophage media decreased threefold but were relatively stable in M2-like macrophage media (Supplementary Fig. S3C). As polarized BMDMs do not divide in culture, this is consistent with greater utilization of glucose in glycolytic-dominant M1-like macrophages. In a separate experiment, iNOS protein levels decreased in M1-like macrophages harvested at later time points associated with lower glucose availability following initial polarization (96 hours). Furthermore, a subtle arginase band was detected 96 hours after initial polarization toward an M1-like phenotype, suggesting a partial shift to an M2-like phenotype as the glucose concentration in the media decreased over time. Conversely, arginase levels were consistently pronounced up to 96 hours after initial polarization toward an M2-like phenotype (Supplementary Fig. S3D). Changes in glucose concentration alone did not result in the polarization of naïve (M0) macrophages (Supplementary Fig. S3D). iNOS mRNA expression was reduced by approximately 50% when M1-like macrophages were cultured in low-glucose conditions compared with high-glucose conditions (Supplementary Fig. S3E), whereas M2-like macrophage CD206 mRNA expression increased under low-glucose conditions after initial polarization. When cultured in low-glucose media (2.5 or 1 mmol/L) for 48 hours, iNOS protein levels were reduced by nearly 90% in M1-like macrophages, whereas arginase levels increased in M2-like macrophages in low-glucose conditions (Supplementary Fig. S3F). M1-like macrophages rely on the generation of reactive oxygen and nitrogen species for their antitumor capabilities (31). Generation of reactive species by M1-like macrophages decreased dramatically in low-glucose conditions (Supplementary Fig. S3G). Together, these data indicate that macrophage polarization and effector function are modulated, at least in part, by glucose availability.

### Glucose is needed to maintain the metabolic profile of M1-like macrophages *in vitro*

**Figure 1.**
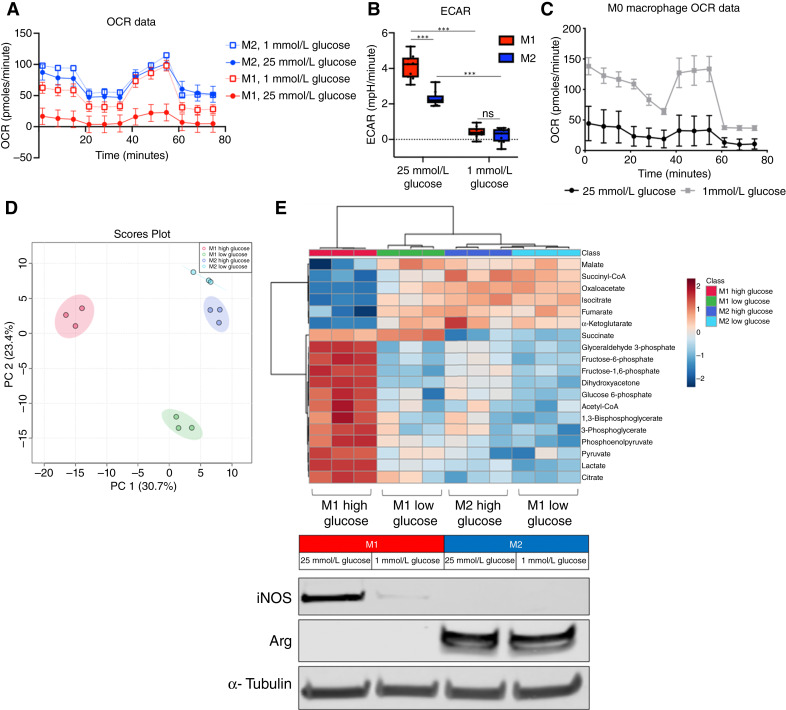
High glucose is needed to maintain the metabolic profile of M1-like macrophages *in vitro*. OCR (**A**) and ECAR (**B**) of M1- and M2-like macrophages at differing glucose levels after 48 hours of treatment, assessed via Seahorse extracellular flux analysis. **C,** OCR of M0 macrophages in high- and low-glucose conditions. **D,** PCA of metabolites from M1- and M2-like macrophages grown in high and low glucose. **E,** LC/MS quantification of glycolytic and tricarboxylic acid cycle metabolites in M1- and M2-like macrophages at indicated glucose concentrations after 48 hours of treatment (top). Corresponding Western blot of iNOS and arginase levels at the time of metabolite harvest are also depicted (bottom), with gel lanes represented in line with the heatmap experimental groups above. ***, *P* < 0.001; ns, not significant.

To explore glucose-dependent macrophage metabolism, Seahorse extracellular flux analysis was performed on M1-like and M2-like macrophages in high- and low-glucose conditions. M2-like macrophages exhibited similar OCRs in both high- and low-glucose conditions (solid and dashed blue lines, respectively, Fig. 1A). M1-like macrophages had a very low OCR in high-glucose conditions (solid red line, Fig. 1A) but exhibited an increase in OCR, partially adopting an M2-like oxidative metabolic profile under low-glucose conditions (dashed red line, Fig. 1A). The ECAR was highest in M1-like macrophages under high-glucose conditions, and they exhibited a reduction in ECAR in low-glucose conditions (Fig. 1B). Naïve macrophages also undergo an increase in OCR under low-glucose conditions (Fig. 1C).

Metabolomic profiling by LC-MS revealed distinct clustering of macrophage metabolic states by polarization and glucose availability. Relative abundances of tricarboxylic acid cycle metabolites were comparable in M2-like macrophages independent of glucose concentration. In contrast, M1-like macrophages underwent a partial metabolic shift from a primarily glycolytic metabolite profile under high glucose to primarily mitochondrial profile under low glucose (Fig. 1D and E). Correspondingly, iNOS protein expression was reduced under low-glucose conditions, whereas arginase expression remained stable in M2-like macrophages [Fig. 1E (bottom)], similar to observations in Supplementary Fig. S3F. These collective data suggest that increased glucose availability is required to maintain the glycolytic metabolic program characteristic of M1-like macrophages.

### Intentional hyperglycemia affects intratumoral glucose levels and immune gene expression in mice

**Figure 2.**
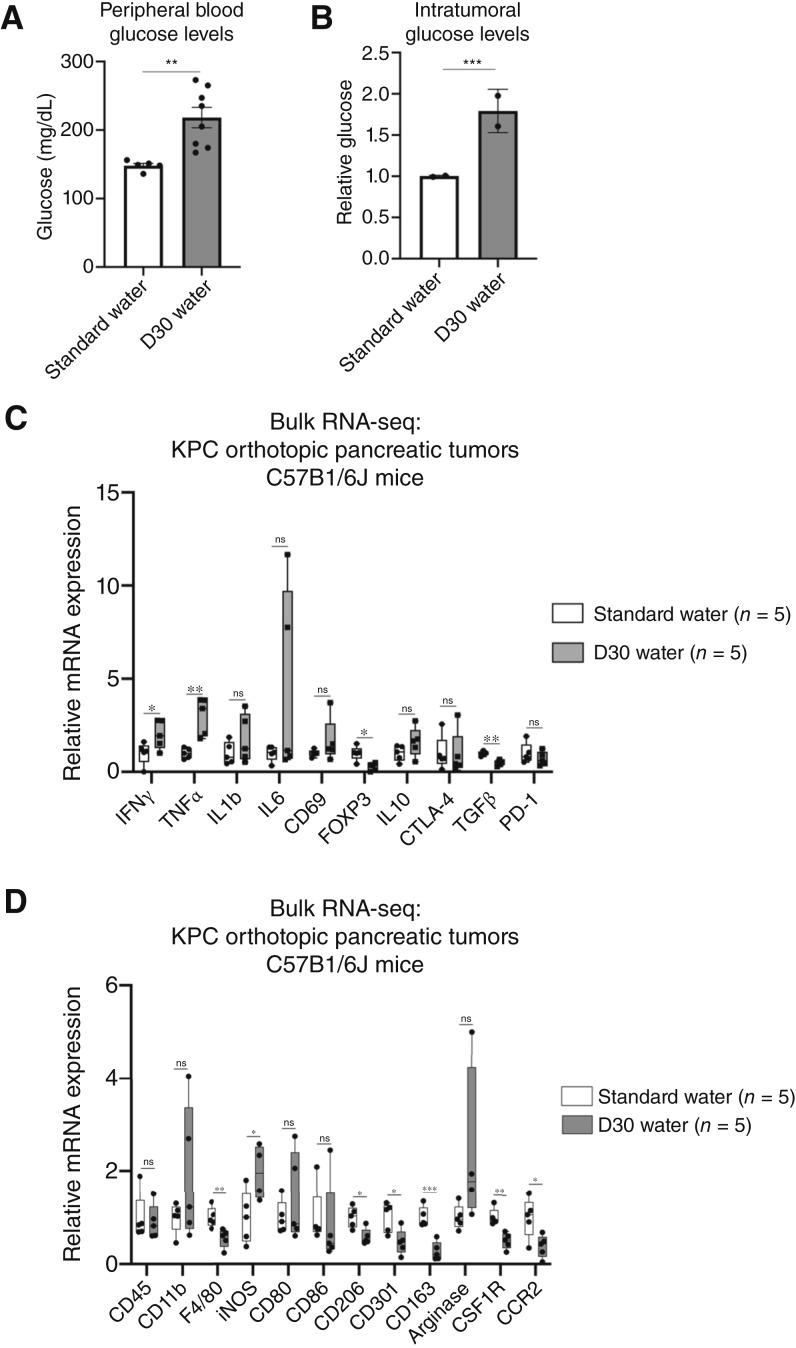
Intentional hyperglycemia affects the TME. **A,** Peripheral glucose levels in tumor-bearing C57Bl/6J mice receiving standard water or D30 water for 2 weeks, measured using an Abbot glucometer. **B,** Relative intratumoral glucose levels from KPC orthotopic pancreatic tumors in C57Bl/6J mice consuming standard water or D30 water for 2 weeks, assessed using a Glucose-Glo assay. **C,** RNA expression of select tumor-suppressor and tumor-promoter genes from treatment-naïve KPC orthotopic pancreatic tumors in C57Bl/6J mice receiving either standard water or D30 water for 4 weeks assessed via bulk RNA-seq. **D,** RNA expression of select immune-related genes from the same tumors as **C**. Single outlying data points were excluded from analyses of D30 iNOS expression and D30 arginase expression (*n* = 4 for these genes). *, *P* < 0.05; **, *P* < 0.01; ***, *P* < 0.001; ns, not significant.

We sought to determine whether macrophage biology was also affected by ambient glucose levels in the PDAC TME *in vivo*. Tumor-bearing C57Bl/6J mice were provided D30 water *ad libitum* to induce short-term hyperglycemia (6). Peripheral blood glucose levels were significantly increased in mice receiving D30 water (Fig. 2A), and this correlated with a near twofold increase in intratumoral glucose levels relative to mice receiving standard water (Fig. 2B). Bulk RNA-seq was performed on orthotopic KPC tumors of immunocompetent mice receiving standard or D30 water (Fig. 2C and D). D30 induced a modest increase in inflammatory genes, such as TNFα and IFNγ, with a corresponding decrease in immunosuppressive genes like FOXP3. General immune and myeloid markers (CD45 and CD11b) were unchanged, yet there were small increases in iNOS and small decreases in other immunosuppression-associated markers (CD206, CD301, and CD163) despite a small decrease in F4/80. Together, these data suggest that increased intratumoral glucose levels may be associated with a more inflammatory and less immunosuppressive TME.

### Macrophage-targeting immunotherapies promote M1-like macrophage polarization *in vitro*

Inhibition of CSF1R decreases the immunosuppressive effects of M2-like macrophages and has been shown to improve the M1:M2 ratio (32, 33). iNOS protein levels of M1-like macrophages were increased under high- and low-glucose conditions with the addition of PLX3397, a CSF1R inhibitor (Supplementary Fig. S4A). In contrast, M2-like macrophage arginase levels decreased with the addition of PLX3397 in both high- and low-glucose conditions. Naïve, M1-like, and M2-like macrophages all express CSF1R (Supplementary Fig. S4B), without significant changes based on glucose levels (Supplementary Fig. S4C). This experiment was replicated using PF-4136309, a CCR2 inhibitor, as inhibition of CCR2 has been shown to also induce polarization toward an M1-like phenotype (34). Again, naïve, M1-like, and M2-like macrophages all expressed CCR2 at baseline (Supplementary Fig. S4B). As with CSF1R inhibition, iNOS levels were increased in M1-like macrophages with the addition of the CCR2 inhibitor, independent of glucose levels (Supplementary Fig. S4D). PF-4136309 did not dramatically affect arginase levels in M2-like macrophages.

PLX3397 and PF-4136309 did not primarily affect macrophage survival under different glucose conditions (Supplementary Fig. S4E), nor did PLX3397 affect KPC growth in high or low glucose, except at exceedingly high drug concentrations (Supplementary Fig. S4F). Thus, under the conditions tested, these drugs do not have a direct impact on macrophage or KPC survival but instead work through an effect on immune subset polarization.

### Increased glucose availability and macrophage-targeting immunotherapies change the immune profile of pancreatic tumors

To further explore how forced hyperglycemia modulates the immune effects of CSF1R and CCR2 inhibition in PDAC, we performed bulk RNA-seq with GO enrichment analysis and flow cytometric profiling in tumors harvested from mice receiving standard or D30 water with or without a macrophage-modulating immunotherapy. Principal component analysis (PCA; Supplementary Fig. S5A) did not show obvious clustering by treatment grouping, and volcano plots (Supplementary Fig. S5B) demonstrated modest differences in gene expression across comparisons.

**Figure 3.**
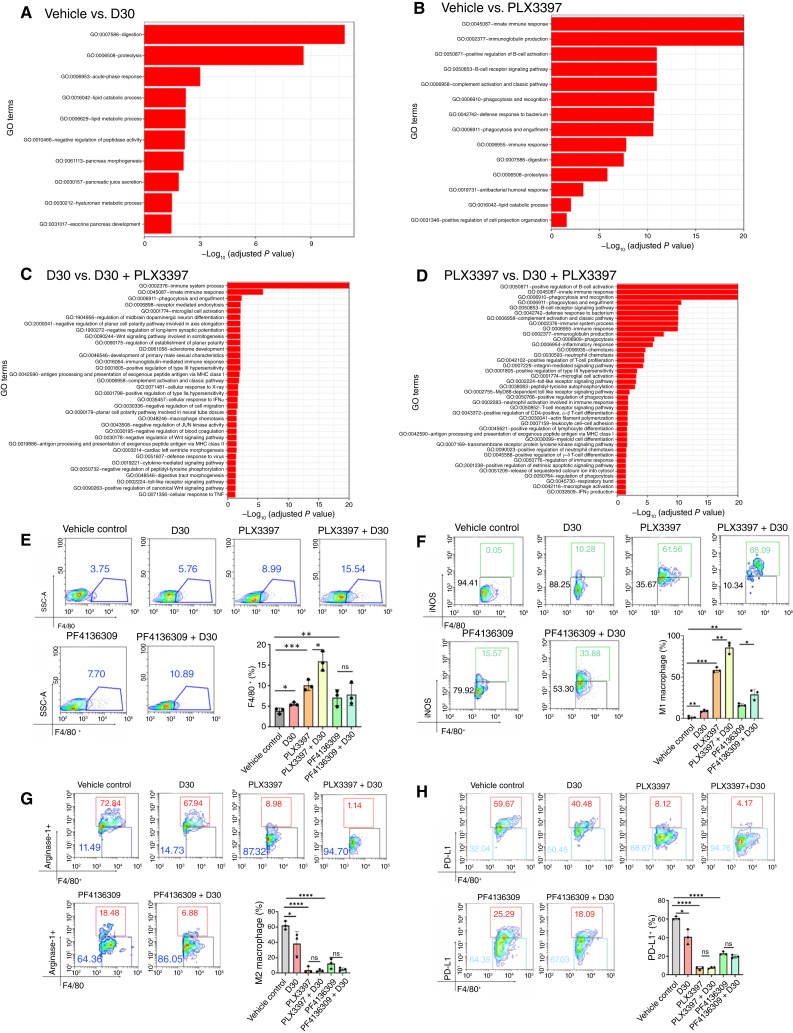
Hyperglycemia affects the immune profile of mice receiving macrophage-modulating immunotherapies. GO analysis of tumors from mice receiving standard water or D30 water with or without PLX3397 (**A–D**). Representative flow cytometry plots and quantitative analyses showing the effects of vehicle control, D30, PLX3397, PLX3397 + D30, PF-4136309, and PF-4136309 + D30 on tumor-associated macrophages and their functional subsets. Tumor-derived single-cell suspensions were gated sequentially on viable cells based on FSC-A and SSC-A properties, followed by the identification of F4/80^+^ macrophages (**E**). Representative contour plots illustrate the frequency of total F4/80^+^ macrophages across treatment groups. Macrophage polarization was further assessed by delineating M1-like macrophages (iNOS^+^; **F**) and M2-like macrophages (arginase-1^+^; **G**). PD-L1 expression on macrophages (**H**) was analyzed within the F4/80^+^ compartment. Bar graphs summarize the percentage of total macrophages, M1-like, M2-like, and PD-L1^+^ macrophage populations for each treatment group. Data are presented as the mean ± SD from independent biological replicates. Statistical significance was assessed using one-way ANOVA with multiple-comparison correction. ns, not significant; *, *P* < 0.05; **, *P* < 0.01; ***, *P* < 0.001; ****, *P* < 0.0001. All gates were defined using fluorescence-minus-one (FMO) controls and applied uniformly across all samples.

Despite only modest gene-level changes and limited separation by PCA, we identified variable differences in pathway-level enrichment based on the treatment administered. In the absence of any immunotherapy, tumors from mice receiving D30 water did not show any enrichment in immune-related pathways relative to standard water (Fig. 3A). In contrast, PLX3397 treatment under normoglycemic conditions led to relative enrichment of immune-associated GO terms, including innate immune response, phagocytosis, and defense responses among others relative to vehicle (Fig. 3B). The addition of PLX3397 to hyperglycemic mice resulted in the enrichment of important immune processes, such as the innate immune response and phagocytosis relative to hyperglycemic mice treated with vehicle (Fig. 3C). PLX3397 delivered in a hyperglycemic context revealed broad immune system activation characterized by innate immune response, phagocytosis, antigen processing and presentation, cytokine-mediated signaling, toll-like receptor signaling, and IFN-associated pathways relative to the drug under normoglycemic conditions (Fig. 3D). These data suggest that D30 supplementation further alters the immune consequences of CSF1R inhibition.

Flow cytometric analysis (gating strategy in Supplementary Fig. S6) revealed that the total F4/80^+^ macrophage abundance was maintained or increased under combined treatment, consistent with macrophage reprogramming rather than depletion (Fig. 3E). PLX3397 treatment significantly increased the proportion of iNOS^+^ F4/80^+^ macrophages while markedly reducing arginase-1^+^ F4/80^+^ macrophages in the TME (Fig. 3F and G). The combination of D30 and PLX3397 produced the highest frequency of iNOS^+^ macrophages and the most pronounced suppression of arginase-1^+^ macrophages. Consistent with reduced immunosuppressive capacity, PD-L1 expression on macrophages was decreased in hyperglycemic mice without additional treatment and reduced further in mice receiving PLX3397 with or without hyperglycemia (Fig. 3H). Similarly, PF-4136309 also had a favorable effect on the immune infiltrate with an increase in iNOS^+^ macrophages and decrease in arginase-1^+^ macrophages (Fig. 3F and G).

PLX3397 and PF-4136309 both significantly increased intratumoral CD3^+^ T-cell abundance, relative to vehicle, with further increases seen with hyperglycemia. This effect was driven primarily by the expansion of CD8^+^ T cells, whereas CD4^+^ T-cell frequencies remained relatively stable (Supplementary Fig. S7). The overall immune cell infiltrate (CD45^+^ cells) was highest in mice receiving a drug combined with hyperglycemia (Supplementary Fig. S8A). Furthermore, fibroblast abundance was reduced among mice treated with PLX3397 or PF-4136309, independent of glucose concentration (Supplementary Fig. S8A), suggesting reduced stromal restriction. B-cell frequencies increased modestly with PLX3397 treatment (Supplementary Fig. S8B), whereas NK1.1^+^ cell abundance was unchanged (Supplementary Fig. S8C), indicating selective immune remodeling.

### Increased glucose availability in the PDAC TME improves macrophage-targeting immunotherapy efficacy

In a subcutaneous tumor model in immunocompetent mice, the combination of PLX3397 and D30 water resulted in a small reduction in tumor growth compared with the monotherapy arms (Supplementary Fig. S9A). This experiment was stopped prematurely, as tumors in the three control arms quickly became ulcerated and threatened mouse wellbeing, whereas the combination treatment arm revealed modest tumor shrinkage. Mice tolerated D30 water without issues and consumed more water relative to euglycemic controls (Supplementary Fig. S9B).

**Figure 4.**
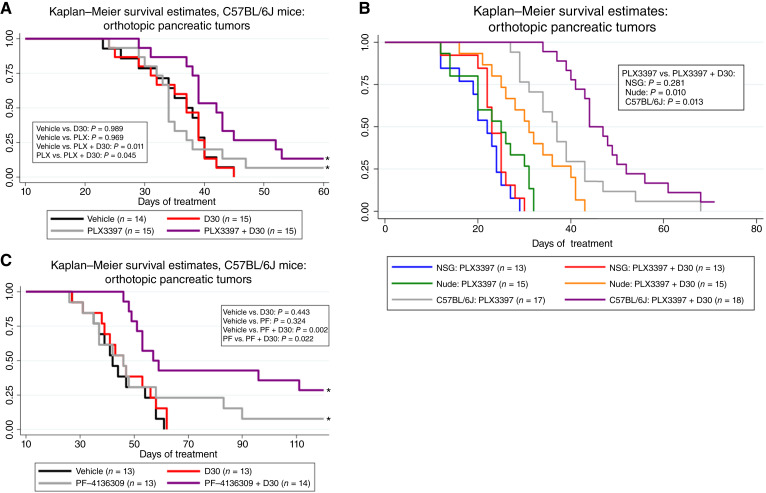
Hyperglycemia improves efficacy of macrophage-targeting immunotherapies *in vivo*. **A,** Overall survival of C57Bl/6J mice with KPC orthotopic tumors treated with vehicle or PLX3397, with or without D30 water. **B,** Overall survival of mice with varying degrees of immunodeficiency (NSG, athymic nude, and C57Bl/6J) treated with PLX3397 with or without D30. **C,** Overall survival of C57Bl/6J mice with KPC orthotopic tumors treated with vehicle or PF-4136309, with or without D30. An asterisk (*) seen in **A** and **C** signifies that mice were alive at the end of the experiment. Median survival was plotted using the Kaplan–Meier method and compared using the log-rank test in the preceding survival experiments.

In an orthotopic model, immunocompetent mice receiving vehicle had nearly identical survival, regardless of whether they consumed standard water or D30 water. Although PLX3397 was ineffective as a monotherapy, there was a modest improvement in overall survival when combined with D30 water. Several mice survived 60 days in the combined modality group (Fig. 4A).

A similar orthotopic survival experiment was performed in diverse mouse models that differ in their immune capabilities. NSG mice, which lack functional T cells, B cells, and NK cells and exhibit impaired cytokine signaling and innate immune function, did not derive additional benefit from combined D30 and PLX3397 compared with PLX3397 alone [Fig. 4B (red and blue curves)]. Athymic nude mice, which are T cell–deficient but retain functional macrophage and innate immune compartments, exhibited a modest survival benefit with combination treatment [Fig. 4B (green and orange curves)], suggesting that T cells are not strictly required but likely contribute to maximal efficacy. A clear survival advantage of combination therapy was observed in immunocompetent C57Bl/6J mice [Fig. 4B (gray and purple curves)], consistent with a model in which macrophage reprogramming is necessary, augmented by intact adaptive immunity. All mouse lineages tolerated treatment (Supplementary Fig. S9C–S9E). Athymic nude and NSG mice experienced similar increases in peripheral glucose levels with D30 water to C57Bl/6J mice (Supplementary Fig. S9F and S9G, similar to Fig. 2A). The survival benefit of combination therapy was also tested using PF-4136309 to independently establish a signal with macrophage modulation under high-glucose conditions, beyond CSF1R inhibition. Again, the drug showed minimal efficacy as a monotherapy but improved overall survival when combined with D30 water, compared with the three control arms (Fig. 4C).

**Figure 5.**
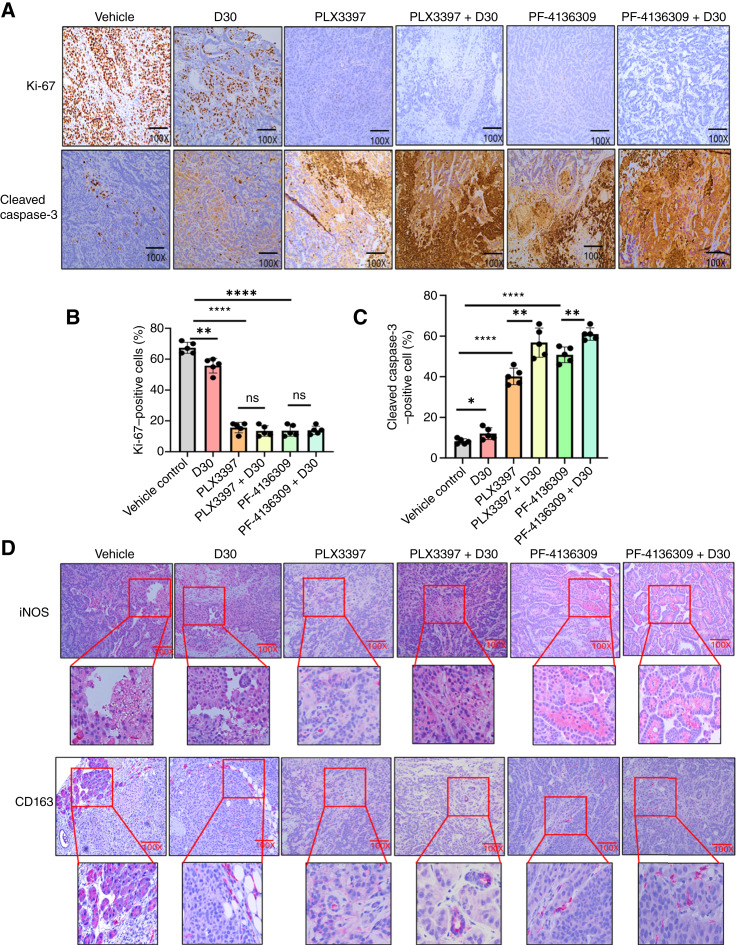
Representative IHC sections of pancreatic tumors from mice treated with vehicle control, D30, PLX3397, PLX3397 + D30, PF-4136309, or PF-4136309 + D30. **A,** Panels depict IHC staining for Ki-67 and cleaved caspase-3 across treatment groups. Brown chromogenic signal indicate positive staining with hematoxylin counterstaining of nuclei. Quantitative analysis of Ki-67 (**B**) and cleaved caspase-3 (**C**). **D,** Panels depict IHC staining for iNOS and CD163 across treatment groups. Magnified views are shows to better demonstrate differences. Pink chromogenic signal indicates positive staining with hematoxylin counterstaining of nuclei. Across treatment groups, qualitative differences in proliferative index, apoptotic activity, and macrophage polarization are evident. Scale bars, 100 μm. All images are representative of multiple tumors examined per group and were acquired using identical staining and imaging conditions. *, *P* < 0.05; **, *P* < 0.01; ****, *P* < 0.0001.

IHC analysis revealed reduced tumor proliferation (Ki-67) and increased apoptosis (cleaved caspase-3) in tumors from mice treated with combination therapy (Fig. 5A–C). There was baseline iNOS staining in tumors treated with vehicle; however, combination therapy was associated with an increased visual density and more broad distribution of iNOS^+^ cells (Fig. 5D). Combination therapy was also associated with a decreased visual density of CD163^+^ cells (Fig. 5D). In summary, these qualitative data align with the aforementioned *in vivo* results and suggest that combination therapy is associated with qualitative changes consistent with reduced tumor proliferation, increased apoptosis, and a relative shift toward an inflammatory phenotype.

Next, we examined the possibility of improved antitumor immune effects of macrophage targeting with hyperglycemia in a hepatic metastases model. Consistent with the above *in vivo* data, the addition of D30 to PLX3397 improved median survival relative to PLX3397 alone (Supplementary Fig. S10A). As with other experiments, mice experienced an increase in peripheral glucose levels with D30 consumption (Supplementary Fig. S10B) and tolerated treatment well (Supplementary Fig. S10C). Two mice in the combined treatment group were found to be without liver tumors after 80 days using bioluminescence imaging and were presumed to be cured after 120 days (Supplementary Fig. S10D and S10E). In the above experiments, mice receiving D30 with PLX3397 exhibited an unexpected and reproducible epiphenomenon of progressive hair depigmentation (Supplementary Fig. S10F), not observed in monotherapy arms, providing indirect evidence of an activated immune effect with the combination.

### A hypothesis-generating case report

A 66-year-old patient with localized PDAC underwent a pancreaticoduodenectomy and was found to have especially aggressive disease intraoperatively (operation performed by senior author, J.M. Winter), with regional metastases observed in 77% of resected lymph nodes. The tumor was microsatellite stable (4). The patient progressed on adjuvant FOLFIRNOX as evidenced by a sharp increase in CA 19-9. The patient was then administered cabiralizumab (CSF1R inhibitor) and nivolumab (PD-1 inhibitor) as part of a clinical trial (NCT02526017). The patient’s CA 19-9 levels dropped precipitously, and the patient survived more than a year after combination immunotherapy was initiated (Supplementary Fig. S11). The patient’s median glucose postoperatively was around 200 mg/dL (calculated HbA1C of 7.8%). Although this observation is purely anecdotal, it is hypothesis-generating and provides clinical context consistent with the preclinical findings. This patient represents one of a small number of exceptional responders to immunotherapy recently described by our group (4).

### Immune profiling of human metastatic PDAC tumors, stratified by glycemic status

**Figure 6.**
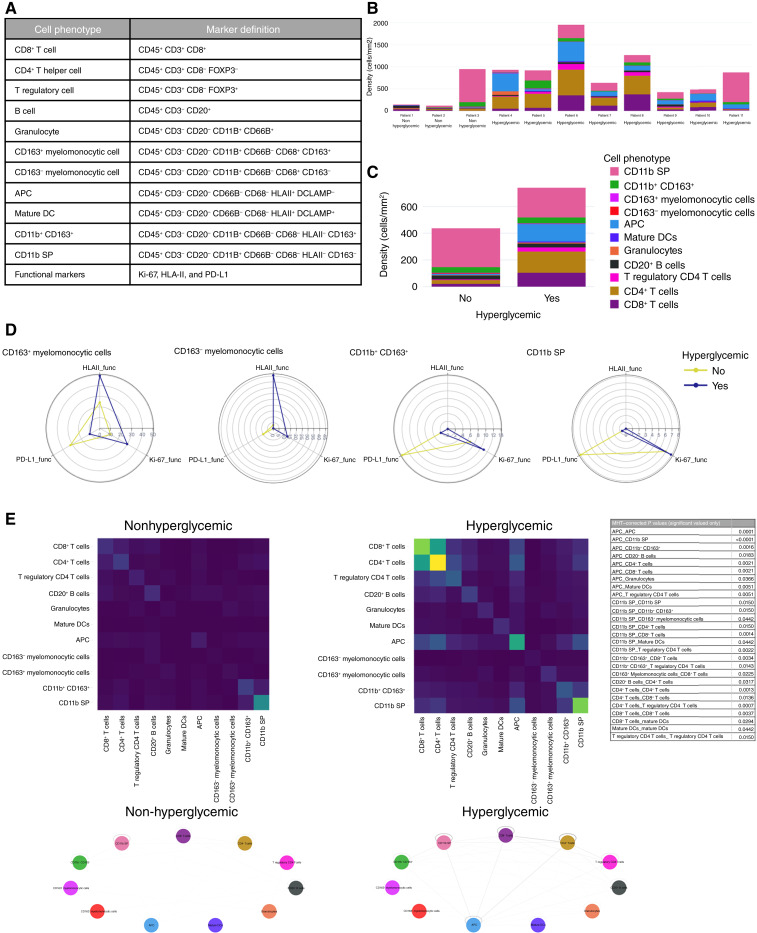
Immune profiling of human metastatic PDAC tumors, stratified by glycemic status. **A,** Phenotypic markers used to define immune cell populations. **B,** Immune cell densities of liver biopsies for all 11 patients (glycemic status of each patient is denoted). **C,** Average of immune cell densities based on glycemic status of the patients (eight hyperglycemic and three nonhyperglycemic). **D,** Radar plots depict the density of cells positive for selected functional markers (HLA-II, Ki-67, and PD-L1) with the indicated myeloid cell populations comparing hyperglycemic (red) and nonhyperglycemic patients (green). **E,** Heatmaps depict normalized frequencies of pairwise spatial proximities between immune cell populations within 20 μm in nonhyperglycemic (left) and hyperglycemic (right) tumors. Corresponding network diagrams illustrate spatial associations. Statistically significant pairwise proximities are listed (*P* < 0.05). *P* values were calculated using a one-sided Mann–Whitney U test and corrected for multiple hypothesis testing using the Benjamini–Hochberg procedure. APC, antigen-presenting cell; DC, dendritic cell; SP, single positive.

mIHC-based (24, 25) spatial profiling (29) was performed on biopsy samples collected from human PDAC liver metastases from normoglycemic and hyperglycemic patients. Immune populations were defined using established phenotypic markers (Fig. 6A). The immune infiltrate varied based on the glycemic status of the patients (Supplementary Fig. S12). Two of the three nonhyperglycemic patients had the lowest overall immune cell density, and the third contained predominately myeloid cells (CD11b single positive; Fig. 6B). When averaged, hyperglycemic patients had a greater density of CD8^+^ T cells, CD4^+^ T cells, and antigen-presenting cells (Fig. 6C). Additionally, hyperglycemic tumors exhibited differences in functional marker expression across select myeloid subsets, including CD163^+^ myelomonocytic cells and CD11b^+^ populations (Fig. 6D). Notably, macrophage populations in the normoglycemic group had higher PD-L1 expression, suggestive of an immunosuppressive phenotype. Spatial proximity analyses further suggested increased immune–immune cell colocalization in hyperglycemic tumors, particularly involving antigen-presenting cells, macrophage subsets, and T cells (Fig. 6E). Given the limited sample size and retrospective nature, these findings are exploratory and correlative.

## Discussion

Broadly effective systemic therapies for patients with PDAC remain elusive. The preponderance of published clinical trial data fails to demonstrate a meaningful clinical benefit of any targeted therapies against PDAC. Erlotinib remains the only targeted therapy approved for all-comers with PDAC, yet median survival only increased by 0.3 months when combined with gemcitabine (35). Olaparib is approved for the 5% of patients with BRCA1/2-mutated PDAC yet did not improve overall survival relative to placebo in a randomized trial (36). Similarly, therapies directed against rare oncogenic drivers, including NTRK gene fusions (∼1%; ref. 37), BRAF V600E mutations (<5%; refs. 38, 39), and KRAS G12C mutations (∼1%; refs. 40, 41), are applicable to a small fraction of patients with PDAC, and survival outcomes have been underwhelming.

Immunotherapies have also been largely ineffective against PDAC (4, 42). Current NCCN guidelines recommend that pembrolizumab (a PD-1 inhibitor) can be offered to the 1% to 2% of patients with PDAC whose tumors exhibit high microsatellite instability (MSI-H; ref. 43). However, past trials yielded an objective response rate of 18% and a median progression-free survival of only 2 months (44). These outcomes are markedly inferior to those of other MSI-H cancers, such as colorectal cancer, in which response rates are as high as 90% (45). Other immune-based strategies like CD40 agonists, cancer vaccines, CAR-T therapies, and oncolytic virus therapies (46, 47) remain investigational.

The CSF1R inhibitor pexidartinib (PLX3397), utilized in the present preclinical study, is FDA-approved for patients with tenosynovial giant cell tumors, which are exceedingly rare (43). Macrophage-targeting immunotherapies have thus far failed to achieve sufficient efficacy in PDAC to warrant clinical adoption. However, small series have identified rare patients with PDAC who derive exceptional benefit from immunotherapies (4, 48–50). Some of these responses have been associated with uncommon genetic abnormalities (e.g., BRCA1, SMAD4, and TSC2) or hereditary conditions (Lynch syndrome). In a multi-institution review of patients with PDAC, 14 exceptional immune responders were identified (4). The median progression-free survival was 12 months, and overall survival was not reached (>2 years). Eight patients were found to have MSI-H tumors, but no unifying driver of response was apparent across the remainder of the cohort. An association between response to therapy and glucose levels was not investigated.

Here, we identify glucose availability within the TME as a previously underappreciated and functionally relevant regulator of immune function in PDAC. More specifically, our data suggest that inflammatory, tumor-suppressive M1-like macrophages are poorly equipped to maintain metabolic fitness and effector function in glucose-limiting conditions likely due to their inherent reliance on glycolysis. Intentional elevation of glucose levels was associated with enhanced antitumor function of M1-like macrophages *in vitro* and *in vivo* and improved the therapeutic efficacy of macrophage-modulating therapies. Moreover, combining intentional hyperglycemia with macrophage-modulating agents more effectively polarized tumor-associated macrophages away from an immunosuppressive M2-like phenotype and toward an inflammatory M1-like phenotype. This combination was associated with a pronounced shift in the M1:M2 macrophage ratio within the TME, accompanied by enhanced CD8^+^ T-cell infiltration and reduced abundance of tumor-associated fibroblasts.

These findings are consistent with emerging literature indicating that systemic metabolic context influences antitumor immunity. Recent work in glioblastoma demonstrated that mice provided with a high-glucose drink exhibited improved overall survival when treated with a PD-1 inhibitor compared with placebo, but the benefit was not observed when mice were provided a control drink or when mice were deficient in CD4^+^ T cells (51). The authors traced the mechanism to proimmune outcomes of the added carbohydrate load to effects on the gut microbiome, whereas our data focus on macrophage metabolism and polarization within tumors. The concept of hyperglycemia promoting a more inflammatory environment has been examined in a diabetic context (i.e., noncancer). Independent investigators have observed that human macrophages polarized toward the inflammatory M1-like phenotype under high-glucose conditions (52). This inflammatory shift is problematic in normal tissues over a long period of time leading to chronic disease yet may be paradoxically beneficial to patients in the context of modifying the TME.

Despite these findings, the translational implications of intentional hyperglycemia warrant careful consideration. Chronic hyperglycemia (i.e., poorly controlled diabetes mellitus) is often associated with a state of immunosuppression. Relative to our current work, macrophages exposed to prolonged hyperglycemia exhibit impaired function (53). However, we underscore that pancreatic tumors are uniquely glucose restricted, and our data specifically address the consequences of restoring glucose availability within the TME over a short time period, rather than modeling chronic and systemic diabetes.

The combination of a PD-1 inhibitor and CSF1R inhibitor was largely ineffective for pancreatic cancer. The aforementioned clinical trial (NCT02526017) was largely interpreted as negative given the 13% objective response rate (54), yet the patient described above had a remarkable treatment response. It is tempting to speculate that immunotherapies like the macrophage-modulating agents used in this study could be potentiated through intentional medical strategies that transiently increase glucose availability, such as deescalation of diabetic medicines, dietary modifications, or glucose infusion therapy.

Finally, we observed a unique phenotype of hair depigmentation in hyperglycemic mice receiving PLX3397. Although not previously reported in murine models of PDAC, hair color changes have been reported in 75% of patients with tenosynovial giant cell tumors treated with pexidartinib (55). In a pooled analysis, the overall response rate among all patients was greater than 60%, and less than 1% of patients exhibited progressive disease, suggesting that pexidartinib is very effective against this particular tumor type. Hair depigmentation may represent a marker of drug efficacy. Interestingly, past studies have shown that M2 macrophages increase melanin production (resulting in darker pigment), whereas M1 macrophages decrease melanin synthesis (56). The side effect of hair depigmentation, seen only in hyperglycemic mice receiving PLX3397, may reflect systemic macrophage reprogramming away from melanin-producing macrophages and could represent a surrogate marker of effective immune polarization. Although speculative, this observation supports the broader concept that the systemic metabolic state can influence macrophage biology and even offer a visual indicator of effective immune modulation.

### Conclusion

In murine models of PDAC, macrophage-targeting immunotherapies showed minimal efficacy as single agents under normoglycemic conditions. Intentionally elevating peripheral and intratumoral glucose levels was associated with enhanced therapeutic efficacy across multiple models. The effect was associated with increased inflammatory M1-like macrophage activity and suppression of tumor-promoting M2-like macrophage programs. These findings identify glucose availability as a key determinant of immunotherapy response and suggest that manipulating the metabolic state may represent a novel strategy to augment immune-based treatments in PDAC.

## Supplementary Material

Supplemental Figure 1 Supplemental Figure 1 - Immune competence is required to restrain pancreatic cancer growth in vivo. A.) Overall survival of mice with different immune backgrounds bearing identical KPC orthotopic pancreatic tumors. NSG mice lack functional T, B, and NK cells and have severely impaired innate immunity including macrophages. Athymic nude mice are deficient in T cells but retain functional innate immune cells including macrophages. C57Bl/6J mice possess an intact adaptive and innate immune system. Mice did not receive any therapy. Median survival was plotted with the Kaplan-Meier method and compared using the log-rank test. Gross photographs of spontaneous liver metastases and primary pancreatic tumor in NSG mice (B.) and normal liver with large primary pancreatic tumor in C57Bl/6J mice (C.).

Supplemental Figure 2 Supplemental Figure 2 - Macrophages differentially affect KPC cell growth based on polarization. Co-culture clonogenic assay of KPC cells with M1- or M2-like bone marrow-derived macrophages. Macrophages without KPC cells are depicted for reference. Relative KPC survival was assessed by quantification of luminescence of luciferase-expressing KPC cells after luciferin supplementation. Background luminescence of macrophages was subtracted. *** indicates p<0.001.

Supplemental Figure 3 Supplemental Figure 3 - Glucose is important for bone marrow-derived macrophage polarization and function in vitro. A.) Relative KPC cell survival when co-cultured with M1- or M2-like macrophages in high vs low glucose, assessed via quantification of KPC cell luminescence (similar experiment as Supplemental Figure 2). Macrophage stimulant controls (LPS and IFN- or IL-4 without BMDM) are depicted for reference. B.) Depiction of baseline KPC growth in 25 mM or 2.5 mM glucose conditions. C.) Measurement of cell culture media glucose concentrations in M1- and M2-like macrophages over a 5-day experiment. Day 0 was defined as when fresh media and macrophage stimulants were added. D.) Western blot of M1- and M2-like macrophages harvested at indicated time points. Cell culture media (initially 25 mM) was not changed during the experiment. Macrophage stimulants (LPS and IFN- or IL-4) were added at timepoint 0 hours and were not re-dosed. Naïve macrophages (M0) are shown for reference. E.) Relative mRNA iNOS and CD206 expression of M0, M1-like, and M2-like macrophages in high and low glucose after 24 hours of culture. F.) Western blot depicting iNOS and arginase protein levels of M1- and M2-like macrophages in indicated glucose concentrations for 48 hours. G.) DCFDA assay showing relative production of reactive oxygen and nitrogen species of M0, M1-, and M2-like macrophages in high and low glucose media. * indicates p<0.05, ** indicates p<0.01, *** indicates p<0.001, ns; not significant.

Supplemental Figure 4 Supplemental Figure 4 - Macrophage-targeting immunotherapies are associated with a protective effect for M1-like macrophage polarization in vitro without impacting macrophage viability. A.) Western blot of M0, M1-, and M2-like macrophages treated with vehicle or PLX3397 (100 nm) in high (25 mM) or low (1 mM) glucose conditions for 48 hours. B.) Baseline western blot of CSF1R and CCR2 in M0, M1-, and M2-like macrophages. C.) Western blot of CSF1R of M1- and M2-like macrophages in high and low glucose conditions. D.) Western blot of M1- and M2-like macrophages treated with vehicle or PF-4136309 (100 nm) in high (25 mM) or low (1 mM) glucose conditions for 48 hours. Protein quantification is normalized to α-Tubulin unless otherwise shown. E.) Bone marrow-derived macrophage survival (M1- and M2-like) treated with varying concentrations of PLX3397 (left) or PF-4136309 (right) in high (25 mM) or low (1 mM) glucose conditions after 4 days assessed via double-stranded DNA quantification (PicoGreen). F.) KPC cell survival treated with varying concentrations of PLX3397 in high (25 mM) or low (2.5 mM) glucose after 4 days assessed via double-stranded DNA quantification (PicoGreen).

Supplemental Figure 5 Supplemental Figure 5 - Principal component analysis (A.) and volcano plots (B.) from bulk RNA-sequencing of orthotopic pancreatic tumors. C57Bl/6J mice bearing KPC orthotopic tumors were randomized to normoglycemic or hyperglycemic conditions. Mice in the hyperglycemia group received D30 drinking water for 8 days prior to initiation of PLX3397 or vehicle treatment, whereas normoglycemic mice received standard drinking water. Tumors were harvested following treatment and subjected to bulk RNA-sequencing. Pairwise comparisons shown include vehicle vs D30, vehicle vs PLX3397, D30 vs combination, and PLX3397 vs combination. Abbreviations: Veh, vehicle; PLX, PLX3397; Combo, D30 plus PLX3397.

Supplemental Figure 6 Supplemental Figure 6 - Flow cytometry gating strategy for immune profiling of pancreatic tumor–bearing mice. Representative flow cytometry plots illustrating the sequential gating strategy used for the identification of lymphoid and myeloid populations in pancreatic tumor samples (indicated as PAN_HG). Briefly, total events were first gated based on forward- and side-scatter (FSC-A vs SSC-A) to exclude debris, followed by singlet discrimination using FSC-A vs FSC-H. Viable leukocytes were identified and gated based on forward- and side-scatter properties, after which CD3^+^ T cells were selected and further subdivided into CD4^+^ and CD8^+^ T cell populations. Macrophages were gated on F4/80^+^ and were further characterized based on expression of iNOS and Arginase-1 to define inflammatory (iNOS^+^) and immunosuppressive (Arginase-1^+^) phenotypes. Additional lineage markers, including NK1.1, B220, CD140α, and PD-L1, were used to delineate specific immune subpopulations as indicated. Percentages shown in each gate represent the proportion of parent populations. All gates were set based on FMO controls and applied uniformly across all samples.

Supplemental Figure 7 Supplemental Figure 7 - Flow cytometric analysis of T cell infiltration following single and combination treatments in pancreatic tumors. Representative flow cytometry plots and quantitative analyses showing the effects of vehicle control, D30, PLX3397, PLX3397 + D30, PF-4136309, and PF-4136309 + D30 on intratumoral T cell populations. Tumor-derived single-cell suspensions were first gated on viable cells based on forward- and side-scatter properties, followed by identification of CD3^+^ T cells. CD3^+^ cells were subsequently subdivided into CD4^+^ and CD8^+^ T cell subsets as indicated. Representative contour plots illustrate the gating strategy and frequencies of each population, with arrows denoting the sequential gating steps. Bar graphs summarize the percentage of CD3^+^, CD4^+^, and CD8^+^ T cells across treatment groups. Data are presented as mean ± S.D. from independent biological replicates. Statistical significance was determined using one-way ANOVA with multiple-comparison correction. ns, not significant; * indicated p<0.05; ** indicated p<0.01; *** indicated p<0.001; **** indicates p<0.0001. All gates were defined using FMO controls and applied consistently across all samples.

Supplemental Figure 8 Supplemental Figure 8 - Flow cytometric analysis of additional immune and stromal cell infiltration following single and combination treatments in pancreatic tumors. A.) Representative flow cytometry plots illustrating the effects of vehicle control, D30, PLX3397, PLX3397 + D30, PF-4136309, and PF-4136309 + D30 on stromal fibroblast populations within pancreatic tumors. Tumor-derived single-cell suspensions were first gated on viable cells based on forward- and side-scatter properties. The CD45^−^ fraction was subsequently analyzed to identify CD140α^+^ fibroblasts, as shown by the indicated gates. Enlarged panels display representative SSC-A versus CD140α plots for each treatment group, with percentages of CD140α^+^ cells indicated. Bar graphs summarize the proportion of CD45^+^ immune cells and CD140α^+^ fibroblasts across treatment groups. B-C.) Representative flow cytometry plots and quantitative analyses showing the impact of vehicle control, D30, PLX3397, PLX3397 + D30, PF-4136309, and PF-4136309 + D30 on intratumoral B cells (B.) and NK1.1+ cells (C.). Representative contour plots display the frequencies of B220^+^ and NK1.1^+^ populations within the parent gates, with percentages shown in each panel. Bar graphs summarize the proportion of B220^+^ B cells and NK1.1^+^ cells across treatment groups. Data are presented as mean ± S.D. from independent biological replicates, with each dot representing an individual tumor. Statistical significance was assessed using one-way ANOVA with multiple-comparison correction. ns, not significant; * indicated p<0.05; ** indicated p<0.01; *** indicated p<0.001. All gates were defined using FMO controls and applied uniformly across all samples.

Supplemental Figure 9 Supplemental Figure 9 - Subcutaneous tumor model, body weight and peripheral glucose levels of mice from experiment in Figure 4B. A.) KPC subcutaneous tumor volumes of C57Bl/6J mice treated with PLX3397 or vehicle with or without D30 water. This experiment was terminated early due to tumor ulceration impacting mouse well-being. Due to the truncated timeframe, tumor volume curves from D30 (red) and PLX3397 (gray) groups are overlapping. B.) Daily water consumption of mice receiving regular drinking water or D30 water. Body weight of C57Bl/6J mice (C.), athymic nude mice (D.), and NSG mice (E.) treated with PLX3397 or PLX3397 in combination with D30 water. Peripheral glucose levels of athymic nude mice (F.) and NSG mice (G.) treated with PLX3397 with or without D30 water after 2 weeks. ** indicates p<0.01, *** indicates p<0.001.

Supplemental Figure 10 Supplemental Figure 10 - Effects of hyperglycemia and PLX3397 in an orthotopic model of hepatic metastases. A.) Median survival of C57Bl/6J mice with orthotopic hepatic metastases treated with PLX3397 with or without D30 water. Treatment was stopped after 60 days. An asterisk (*) indicates two mice in the combination group were euthanized after surviving 120 days. Photograph insert depicts gross appearance of multifocal hepatic metastases after splenic injection. B.) Peripheral glucose levels of mice bearing orthotopic hepatic metastases after 2 weeks of treatment. C.) Body weights throughout the study period. D.) Bioluminescence imaging of mice (n=3) treated with PLX3397 and D30 water alive at day 90. E.) Gross depiction of mice (n=2) after euthanasia (day 120) did not demonstrate evidence of hepatic metastases (left and middle). Gross depiction of mouse euthanized on day 95 due to signs of severe systemic illness demonstrates a large tumor in the right lobe of the liver, consistent with bioluminescence imaging (right). F.) Mice receiving PLX3397 combined with D30 develop progressive fur depigmentation.

Supplemental Figure 11 Supplemental Figure 11 - Durable response to immunotherapy in a patient with diabetes who developed metastatic disease after a pancreatectomy and progressed on chemotherapy. CA 19-9 (U/mL) values are depicted by the red line. Peripheral glucose levels (mg/dL) are depicted by the black line. The patient’s median glucose level over the course of treatment is depicted with the horizontal gray dashed line (190 mg/dL).Supplemental Figure 11 - Durable response to immunotherapy in a patient with diabetes who developed metastatic disease after a pancreatectomy and progressed on chemotherapy. CA 19-9 (U/mL) values are depicted by the red line. Peripheral glucose levels (mg/dL) are depicted by the black line. The patient’s median glucose level over the course of treatment is depicted with the horizontal gray dashed line (190 mg/dL).

Supplemental Figure 12 Supplemental Figure 12 - Hyperglycemia is associated with an altered immune profile. Representative mIHC and immunofluorescence images of metastatic PDAC tumors obtained from non-hyperglycemic (left) and hyperglycemic (right) patients treated at OHSU. Single stained IHC images (top) depict tumor cells (PanCK), antigen-presenting cells (HLA-II), T cells (CD3), myeloid cells (CD 11b, CD163), and dendritic cells (CD11c). Immunofluorescence images (bottom) illustrate spatial relationships between cell populations in the tumor microenvironment. Scale bars, 250 µm.

## Data Availability

Data generated in this study are available upon request to the corresponding author. LC-/MS data have been deposited to the UCSD Metabolomics Workbench (https://www.metabolomicsworkbench.org; Study ID ST004822; http://dx.doi.org/10.21228/M8ZZ88). Bulk RNA-seq data have been submitted to the NIH International Nucleotide Sequence Database Collection (https://www.ncbi.nlm.nih.gov/sra/PRJNA1460891, BioProject: PRJNA1460891).
