# Supplementary figures and images for: Increased Glucose Availability Sensitizes Pancreatic Cancer to Macrophage-Targeting Immunotherapies
Source: Cancer Res Commun. 2026 Jun 23;6(6):1470–85. doi: 10.1158/2767-9764.CRC-25-0338 (PMC13288262; doi:10.1158/2767-9764.CRC-25-0338)

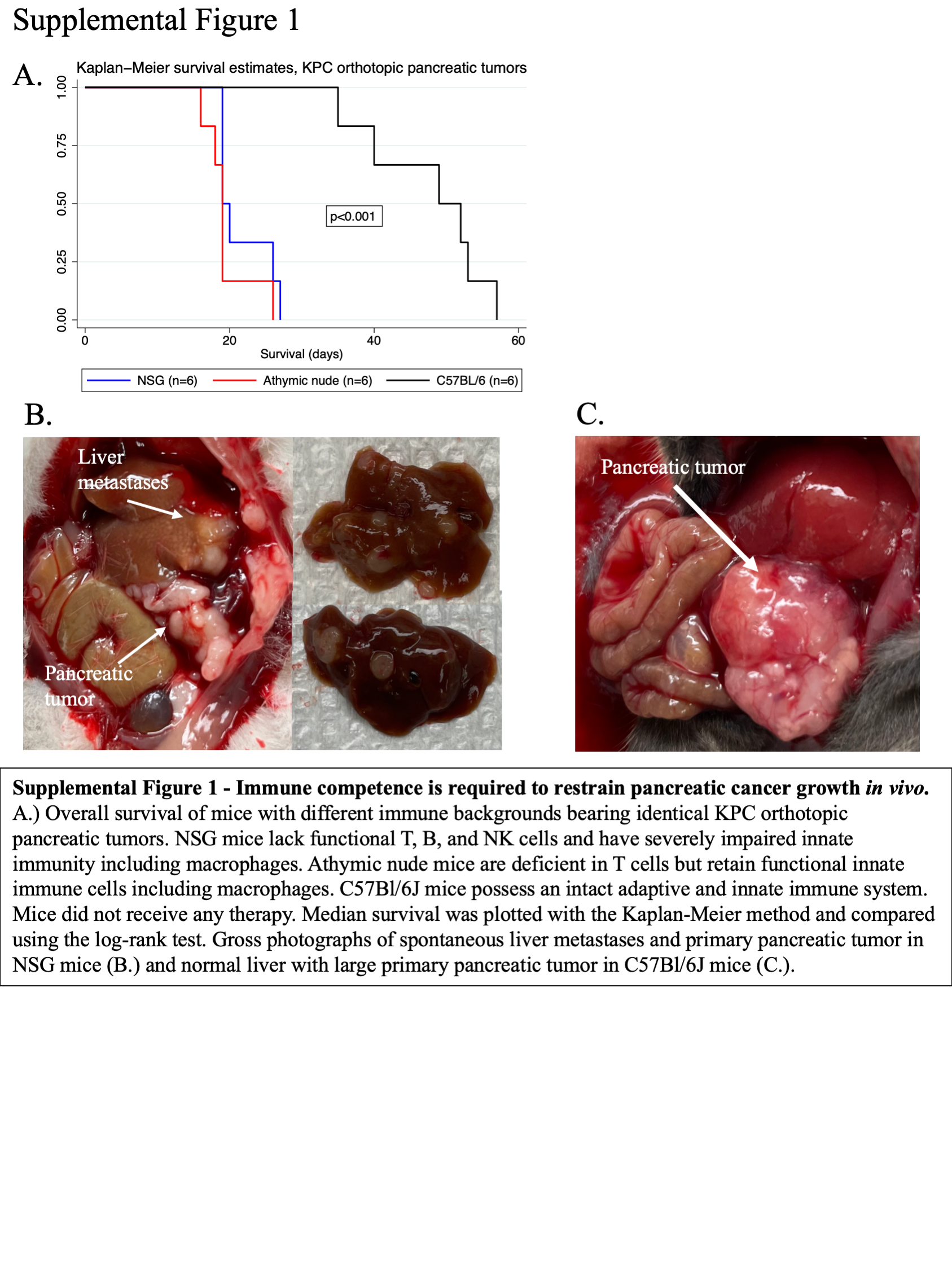

Supplement: Supplemental Figure 1 — Immune competence is required to restrain pancreatic cancer growth in vivo. A.) Overall survival of mice with different immune backgrounds bearing identical KPC orthotopic pancreatic tumors. NSG mice lack functional T, B, and NK cells and have severely impaired innate immunity including macrophages. Athymic nude mice are deficient in T cells but retain functional innate immune cells including macrophages. C57Bl/6J mice possess an intact adaptive and innate immune system. Mice did not receive any therapy. Median survival was plotted with the Kaplan-Meier method and compared using the log-rank test. Gross photographs of spontaneous liver metastases and primary pancreatic tumor in NSG mice (B.) and normal liver with large primary pancreatic tumor in C57Bl/6J mice (C.). [file crc-25-0338_supplemental_figure_1_suppsf1.png]

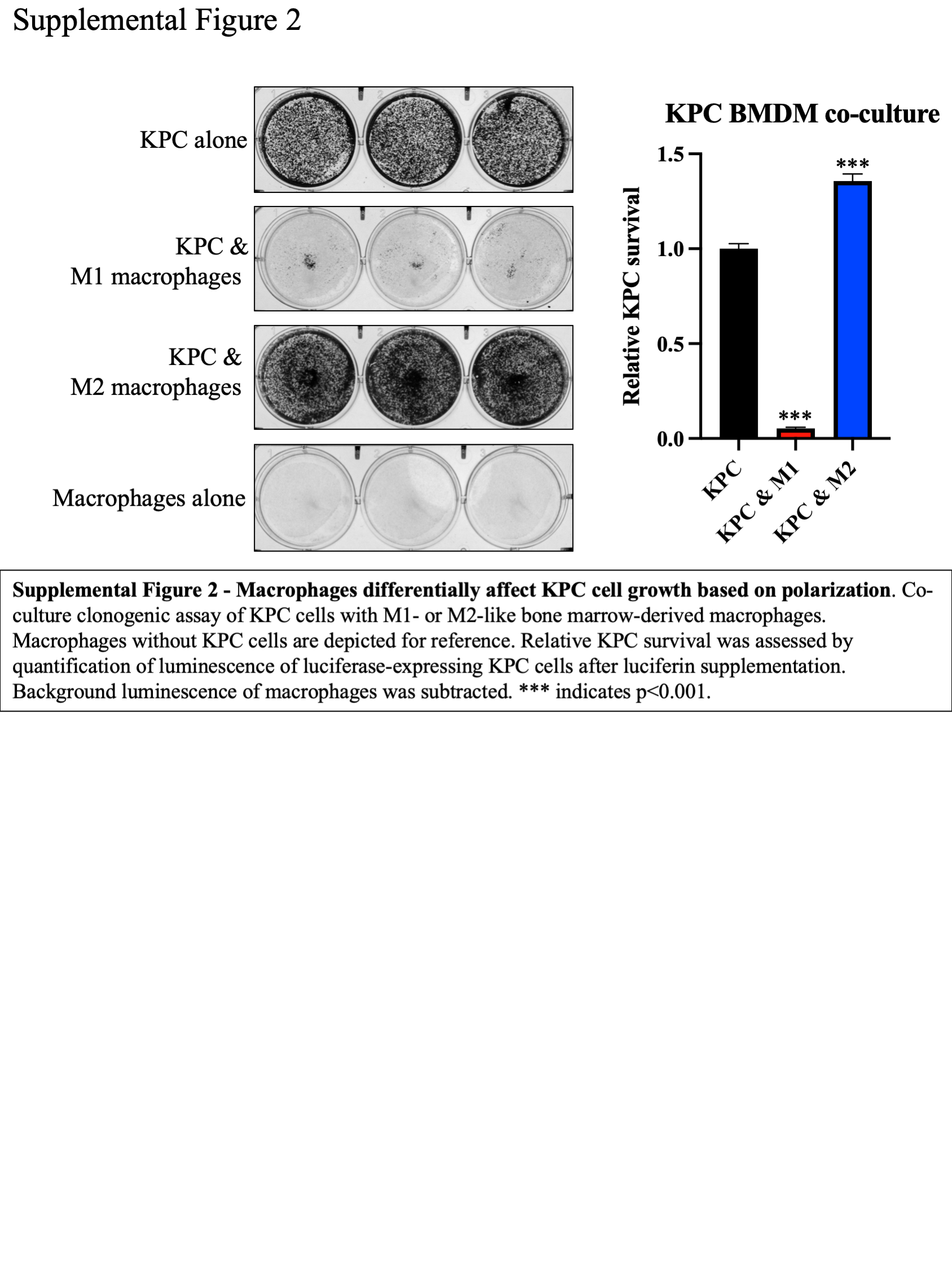

Supplement: Supplemental Figure 2 — Macrophages differentially affect KPC cell growth based on polarization. Co-culture clonogenic assay of KPC cells with M1- or M2-like bone marrow-derived macrophages. Macrophages without KPC cells are depicted for reference. Relative KPC survival was assessed by quantification of luminescence of luciferase-expressing KPC cells after luciferin supplementation. Background luminescence of macrophages was subtracted. *** indicates p<0.001. [file crc-25-0338_supplemental_figure_2_suppsf2.png]

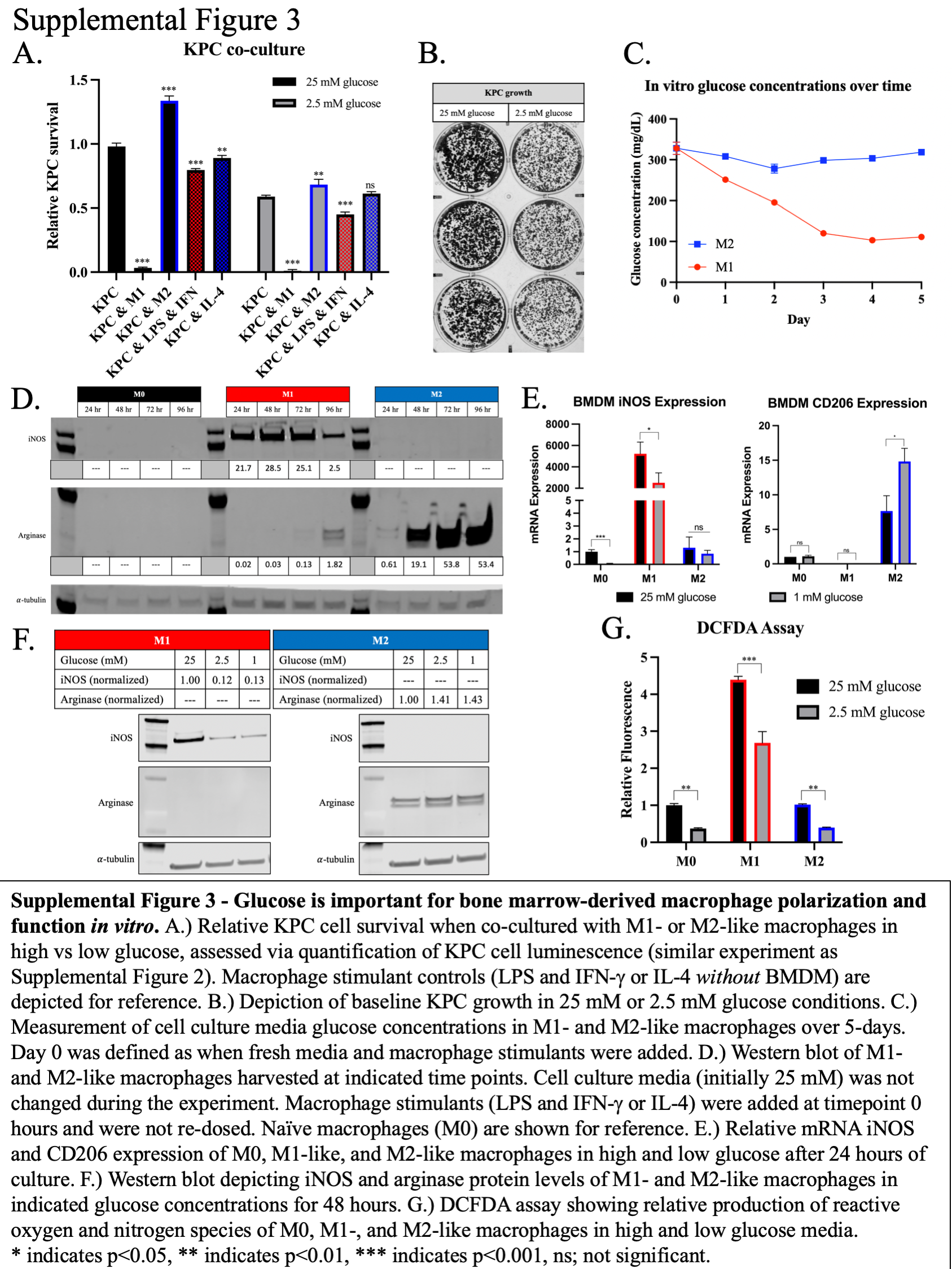

Supplement: Supplemental Figure 3 — Glucose is important for bone marrow-derived macrophage polarization and function in vitro. A.) Relative KPC cell survival when co-cultured with M1- or M2-like macrophages in high vs low glucose, assessed via quantification of KPC cell luminescence (similar experiment as Supplemental Figure 2). Macrophage stimulant controls (LPS and IFN- or IL-4 without BMDM) are depicted for reference. B.) Depiction of baseline KPC growth in 25 mM or 2.5 mM glucose conditions. C.) Measurement of cell culture media glucose concentrations in M1- and M2-like macrophages over a 5-day experiment. Day 0 was defined as when fresh media and macrophage stimulants were added. D.) Western blot of M1- and M2-like macrophages harvested at indicated time points. Cell culture media (initially 25 mM) was not changed during the experiment. Macrophage stimulants (LPS and IFN- or IL-4) were added at timepoint 0 hours and were not re-dosed. Naïve macrophages (M0) are shown for reference. E.) Relative mRNA iNOS and CD206 expression of M0, M1-like, and M2-like macrophages in high and low glucose after 24 hours of culture. F.) Western blot depicting iNOS and arginase protein levels of M1- and M2-like macrophages in indicated glucose concentrations for 48 hours. G.) DCFDA assay showing relative production of reactive oxygen and nitrogen species of M0, M1-, and M2-like macrophages in high and low glucose media. * indicates p<0.05, ** indicates p<0.01, *** indicates p<0.001, ns; not significant. [file crc-25-0338_supplemental_figure_3_suppsf3.png]

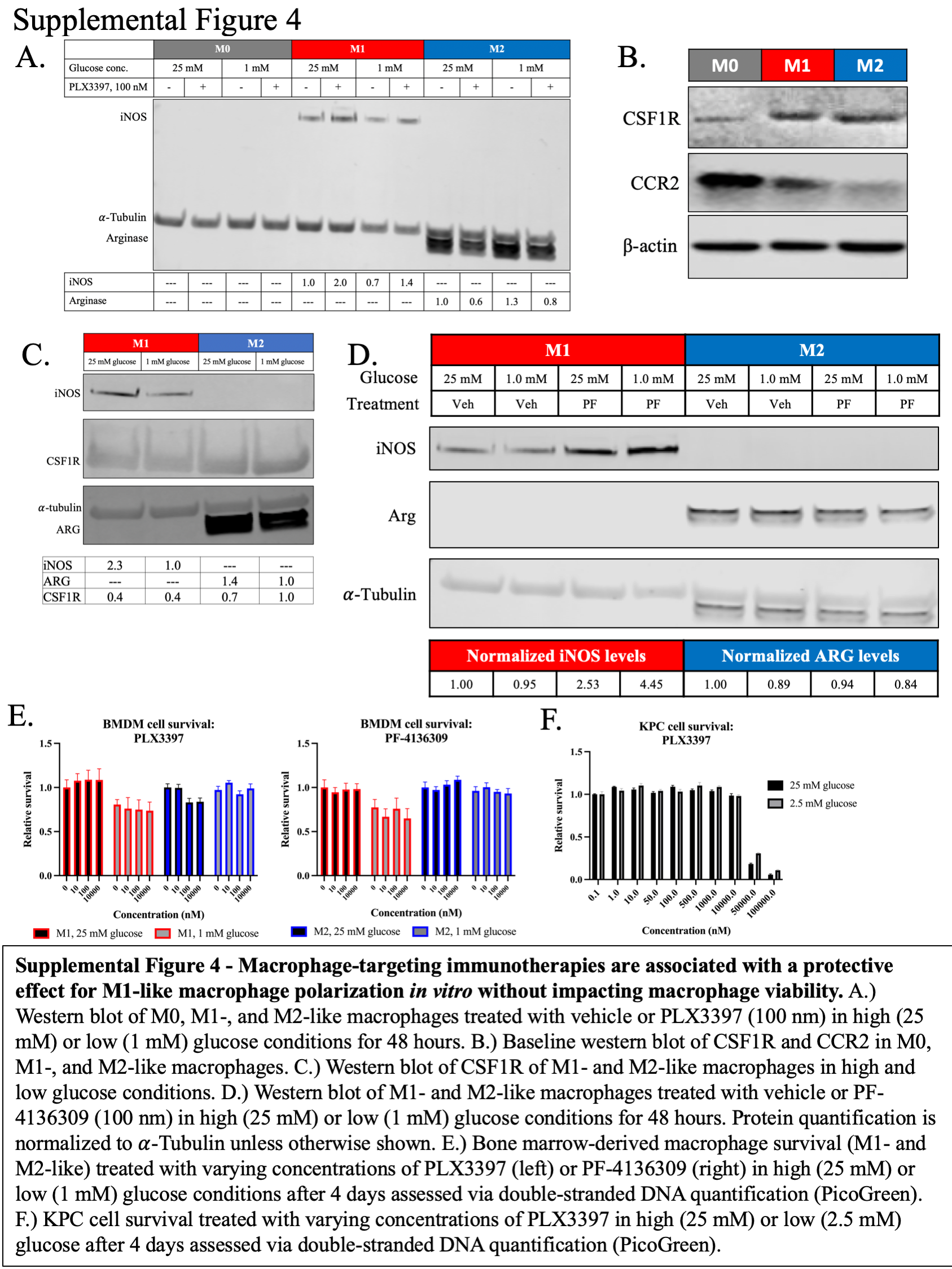

Supplement: Supplemental Figure 4 — Macrophage-targeting immunotherapies are associated with a protective effect for M1-like macrophage polarization in vitro without impacting macrophage viability. A.) Western blot of M0, M1-, and M2-like macrophages treated with vehicle or PLX3397 (100 nm) in high (25 mM) or low (1 mM) glucose conditions for 48 hours. B.) Baseline western blot of CSF1R and CCR2 in M0, M1-, and M2-like macrophages. C.) Western blot of CSF1R of M1- and M2-like macrophages in high and low glucose conditions. D.) Western blot of M1- and M2-like macrophages treated with vehicle or PF-4136309 (100 nm) in high (25 mM) or low (1 mM) glucose conditions for 48 hours. Protein quantification is normalized to α-Tubulin unless otherwise shown. E.) Bone marrow-derived macrophage survival (M1- and M2-like) treated with varying concentrations of PLX3397 (left) or PF-4136309 (right) in high (25 mM) or low (1 mM) glucose conditions after 4 days assessed via double-stranded DNA quantification (PicoGreen). F.) KPC cell survival treated with varying concentrations of PLX3397 in high (25 mM) or low (2.5 mM) glucose after 4 days assessed via double-stranded DNA quantification (PicoGreen). [file crc-25-0338_supplemental_figure_4_suppsf4.png]

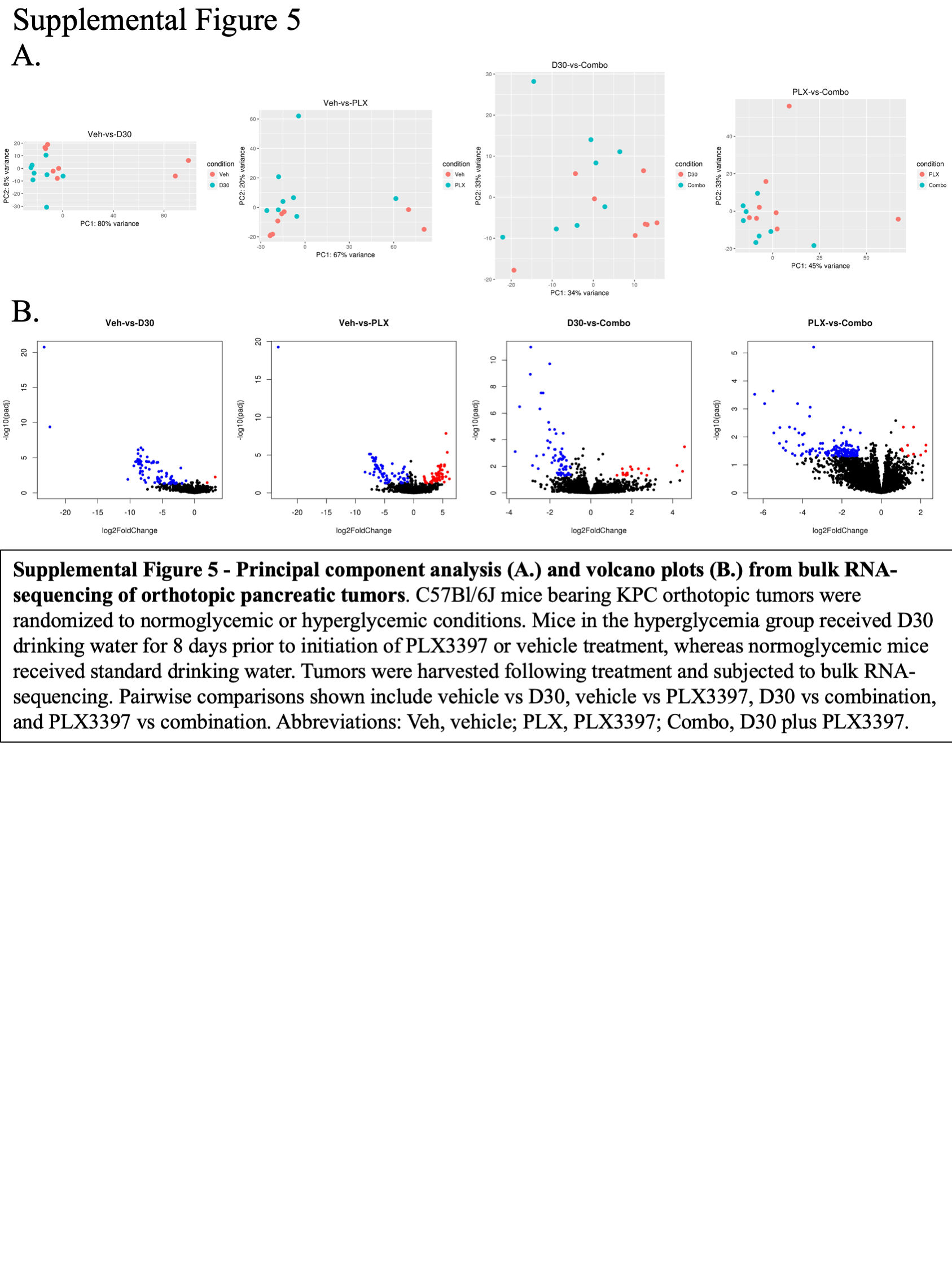

Supplement: Supplemental Figure 5 — Principal component analysis (A.) and volcano plots (B.) from bulk RNA-sequencing of orthotopic pancreatic tumors. C57Bl/6J mice bearing KPC orthotopic tumors were randomized to normoglycemic or hyperglycemic conditions. Mice in the hyperglycemia group received D30 drinking water for 8 days prior to initiation of PLX3397 or vehicle treatment, whereas normoglycemic mice received standard drinking water. Tumors were harvested following treatment and subjected to bulk RNA-sequencing. Pairwise comparisons shown include vehicle vs D30, vehicle vs PLX3397, D30 vs combination, and PLX3397 vs combination. Abbreviations: Veh, vehicle; PLX, PLX3397; Combo, D30 plus PLX3397. [file crc-25-0338_supplemental_figure_5_suppsf5.png]

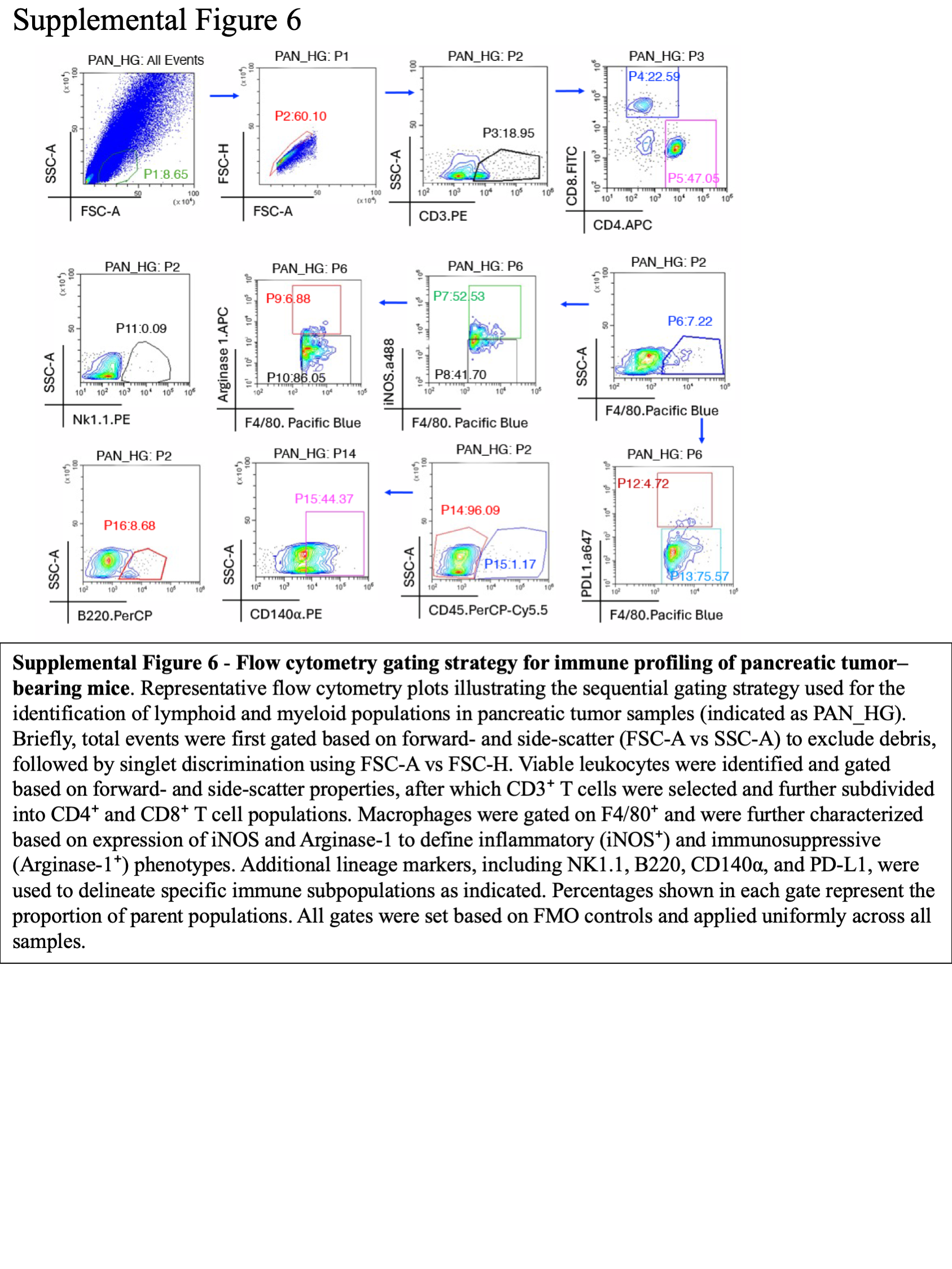

Supplement: Supplemental Figure 6 — Flow cytometry gating strategy for immune profiling of pancreatic tumor–bearing mice. Representative flow cytometry plots illustrating the sequential gating strategy used for the identification of lymphoid and myeloid populations in pancreatic tumor samples (indicated as PAN_HG). Briefly, total events were first gated based on forward- and side-scatter (FSC-A vs SSC-A) to exclude debris, followed by singlet discrimination using FSC-A vs FSC-H. Viable leukocytes were identified and gated based on forward- and side-scatter properties, after which CD3+ T cells were selected and further subdivided into CD4+ and CD8+ T cell populations. Macrophages were gated on F4/80+ and were further characterized based on expression of iNOS and Arginase-1 to define inflammatory (iNOS+) and immunosuppressive (Arginase-1+) phenotypes. Additional lineage markers, including NK1.1, B220, CD140α, and PD-L1, were used to delineate specific immune subpopulations as indicated. Percentages shown in each gate represent the proportion of parent populations. All gates were set based on FMO controls and applied uniformly across all samples. [file crc-25-0338_supplemental_figure_6_suppsf6.png]

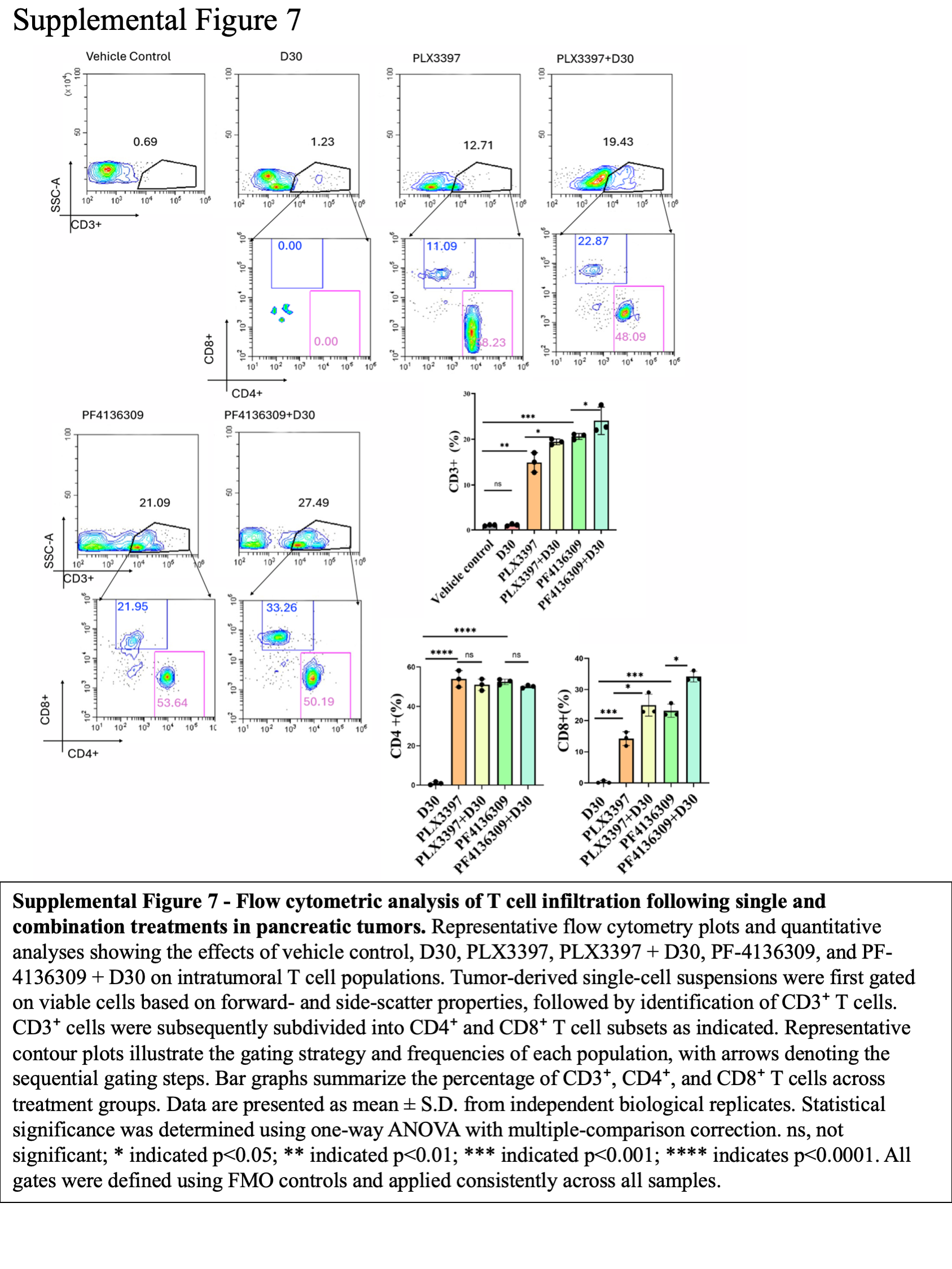

Supplement: Supplemental Figure 7 — Flow cytometric analysis of T cell infiltration following single and combination treatments in pancreatic tumors. Representative flow cytometry plots and quantitative analyses showing the effects of vehicle control, D30, PLX3397, PLX3397 + D30, PF-4136309, and PF-4136309 + D30 on intratumoral T cell populations. Tumor-derived single-cell suspensions were first gated on viable cells based on forward- and side-scatter properties, followed by identification of CD3+ T cells. CD3+ cells were subsequently subdivided into CD4+ and CD8+ T cell subsets as indicated. Representative contour plots illustrate the gating strategy and frequencies of each population, with arrows denoting the sequential gating steps. Bar graphs summarize the percentage of CD3+, CD4+, and CD8+ T cells across treatment groups. Data are presented as mean ± S.D. from independent biological replicates. Statistical significance was determined using one-way ANOVA with multiple-comparison correction. ns, not significant; * indicated p<0.05; ** indicated p<0.01; *** indicated p<0.001; **** indicates p<0.0001. All gates were defined using FMO controls and applied consistently across all samples. [file crc-25-0338_supplemental_figure_7_suppsf7.png]

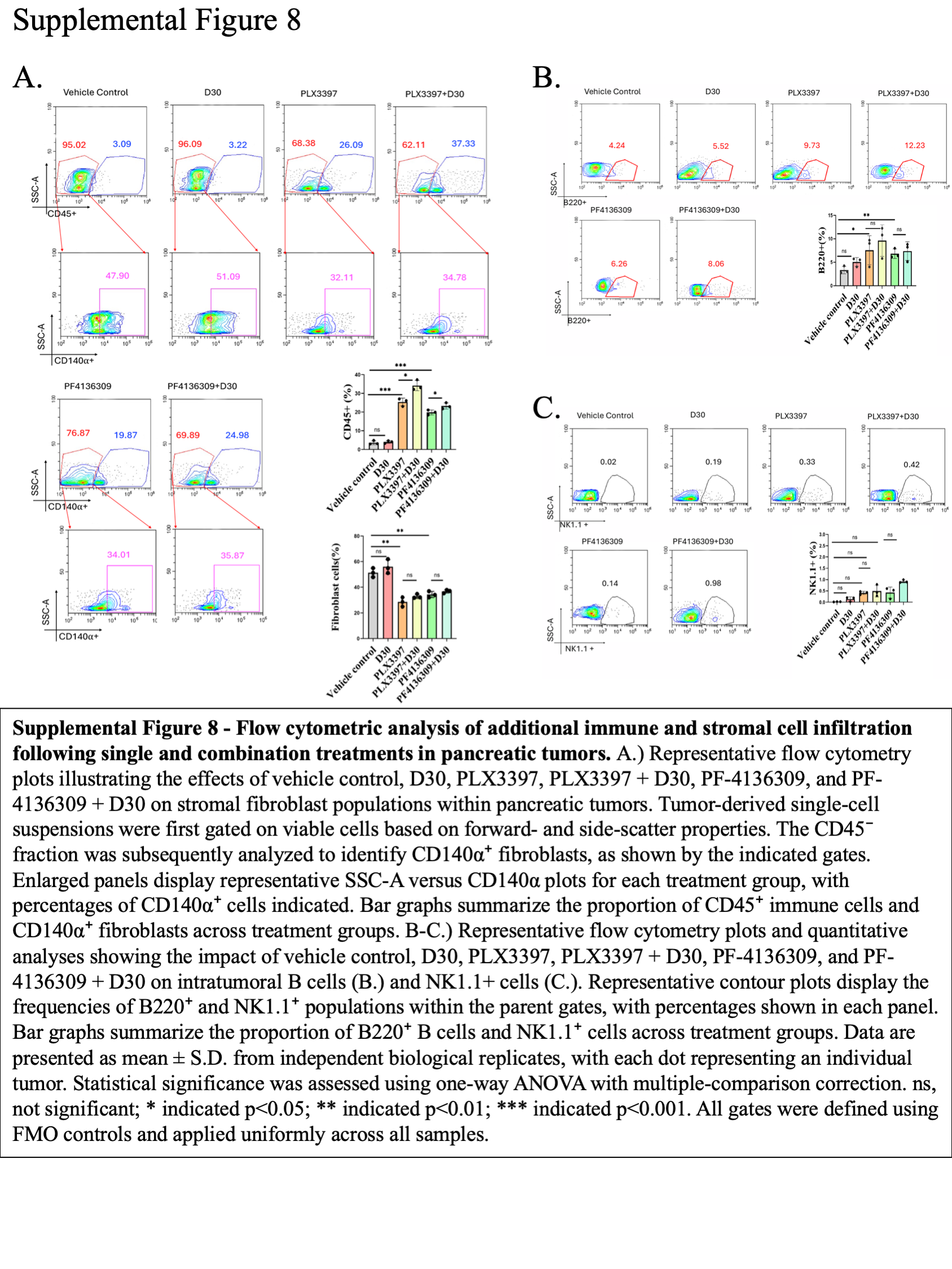

Supplement: Supplemental Figure 8 — Flow cytometric analysis of additional immune and stromal cell infiltration following single and combination treatments in pancreatic tumors. A.) Representative flow cytometry plots illustrating the effects of vehicle control, D30, PLX3397, PLX3397 + D30, PF-4136309, and PF-4136309 + D30 on stromal fibroblast populations within pancreatic tumors. Tumor-derived single-cell suspensions were first gated on viable cells based on forward- and side-scatter properties. The CD45− fraction was subsequently analyzed to identify CD140α+ fibroblasts, as shown by the indicated gates. Enlarged panels display representative SSC-A versus CD140α plots for each treatment group, with percentages of CD140α+ cells indicated. Bar graphs summarize the proportion of CD45+ immune cells and CD140α+ fibroblasts across treatment groups. B-C.) Representative flow cytometry plots and quantitative analyses showing the impact of vehicle control, D30, PLX3397, PLX3397 + D30, PF-4136309, and PF-4136309 + D30 on intratumoral B cells (B.) and NK1.1+ cells (C.). Representative contour plots display the frequencies of B220+ and NK1.1+ populations within the parent gates, with percentages shown in each panel. Bar graphs summarize the proportion of B220+ B cells and NK1.1+ cells across treatment groups. Data are presented as mean ± S.D. from independent biological replicates, with each dot representing an individual tumor. Statistical significance was assessed using one-way ANOVA with multiple-comparison correction. ns, not significant; * indicated p<0.05; ** indicated p<0.01; *** indicated p<0.001. All gates were defined using FMO controls and applied uniformly across all samples. [file crc-25-0338_supplemental_figure_8_suppsf8.png]

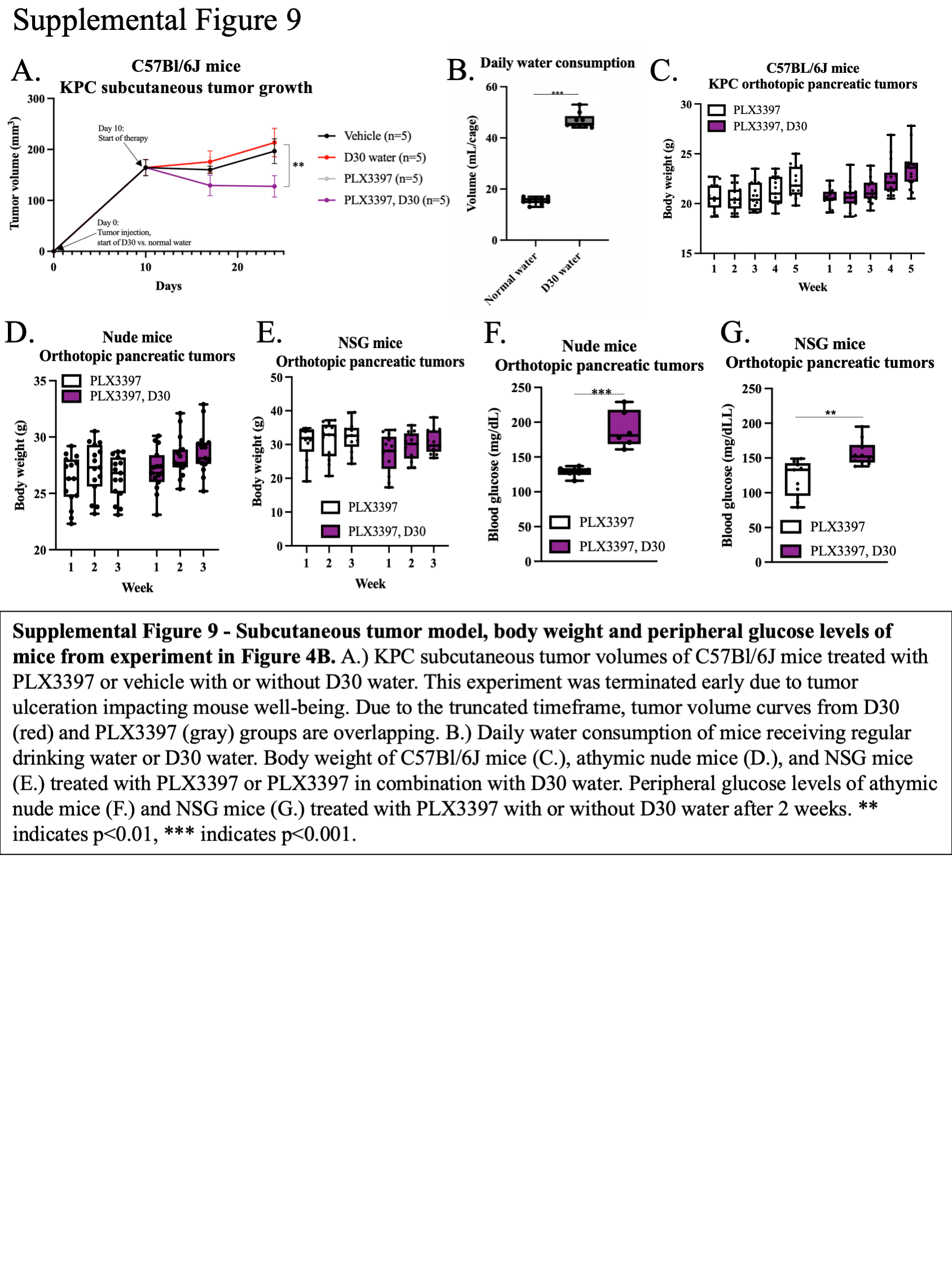

Supplement: Supplemental Figure 9 — Subcutaneous tumor model, body weight and peripheral glucose levels of mice from experiment in Figure 4B. A.) KPC subcutaneous tumor volumes of C57Bl/6J mice treated with PLX3397 or vehicle with or without D30 water. This experiment was terminated early due to tumor ulceration impacting mouse well-being. Due to the truncated timeframe, tumor volume curves from D30 (red) and PLX3397 (gray) groups are overlapping. B.) Daily water consumption of mice receiving regular drinking water or D30 water. Body weight of C57Bl/6J mice (C.), athymic nude mice (D.), and NSG mice (E.) treated with PLX3397 or PLX3397 in combination with D30 water. Peripheral glucose levels of athymic nude mice (F.) and NSG mice (G.) treated with PLX3397 with or without D30 water after 2 weeks. ** indicates p<0.01, *** indicates p<0.001. [file crc-25-0338_supplemental_figure_9_suppsf9.png]

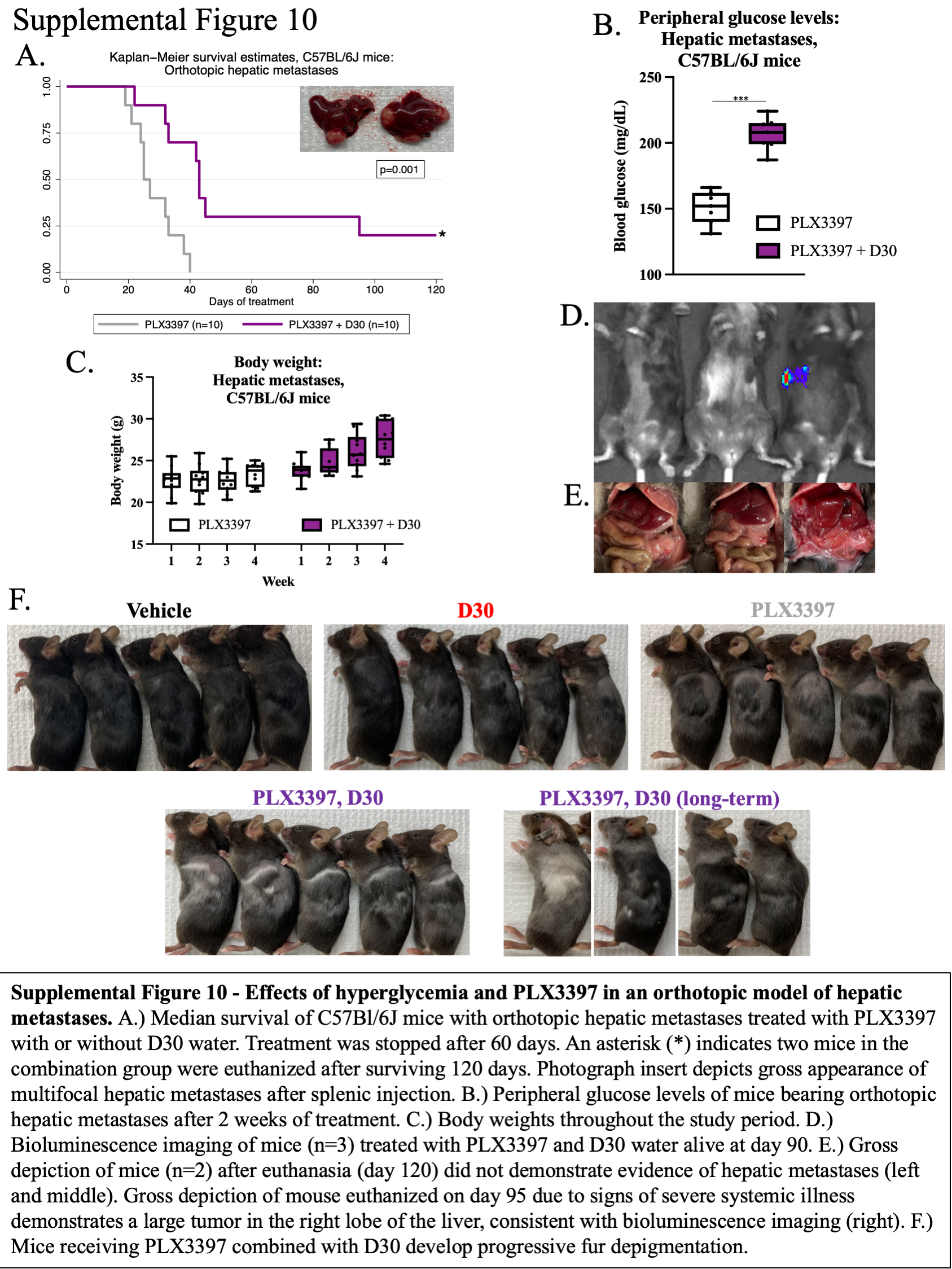

Supplement: Supplemental Figure 10 — Effects of hyperglycemia and PLX3397 in an orthotopic model of hepatic metastases. A.) Median survival of C57Bl/6J mice with orthotopic hepatic metastases treated with PLX3397 with or without D30 water. Treatment was stopped after 60 days. An asterisk (*) indicates two mice in the combination group were euthanized after surviving 120 days. Photograph insert depicts gross appearance of multifocal hepatic metastases after splenic injection. B.) Peripheral glucose levels of mice bearing orthotopic hepatic metastases after 2 weeks of treatment. C.) Body weights throughout the study period. D.) Bioluminescence imaging of mice (n=3) treated with PLX3397 and D30 water alive at day 90. E.) Gross depiction of mice (n=2) after euthanasia (day 120) did not demonstrate evidence of hepatic metastases (left and middle). Gross depiction of mouse euthanized on day 95 due to signs of severe systemic illness demonstrates a large tumor in the right lobe of the liver, consistent with bioluminescence imaging (right). F.) Mice receiving PLX3397 combined with D30 develop progressive fur depigmentation. [file crc-25-0338_supplemental_figure_10_suppsf10.png]

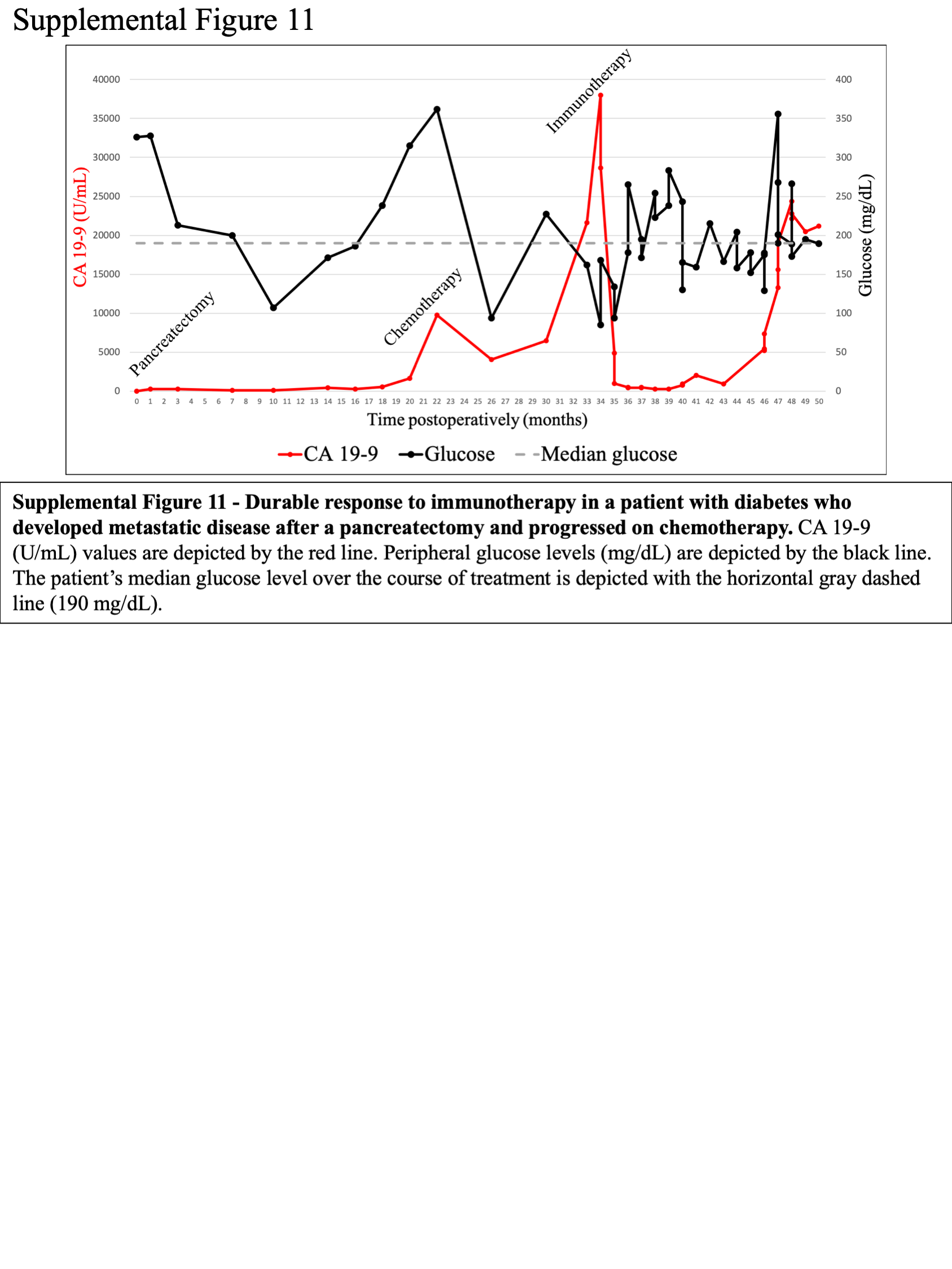

Supplement: Supplemental Figure 11 — Durable response to immunotherapy in a patient with diabetes who developed metastatic disease after a pancreatectomy and progressed on chemotherapy. CA 19-9 (U/mL) values are depicted by the red line. Peripheral glucose levels (mg/dL) are depicted by the black line. The patient’s median glucose level over the course of treatment is depicted with the horizontal gray dashed line (190 mg/dL).Supplemental Figure 11 - Durable response to immunotherapy in a patient with diabetes who developed metastatic disease after a pancreatectomy and progressed on chemotherapy. CA 19-9 (U/mL) values are depicted by the red line. Peripheral glucose levels (mg/dL) are depicted by the black line. The patient’s median glucose level over the course of treatment is depicted with the horizontal gray dashed line (190 mg/dL). [file crc-25-0338_supplemental_figure_11_suppsf11.png]

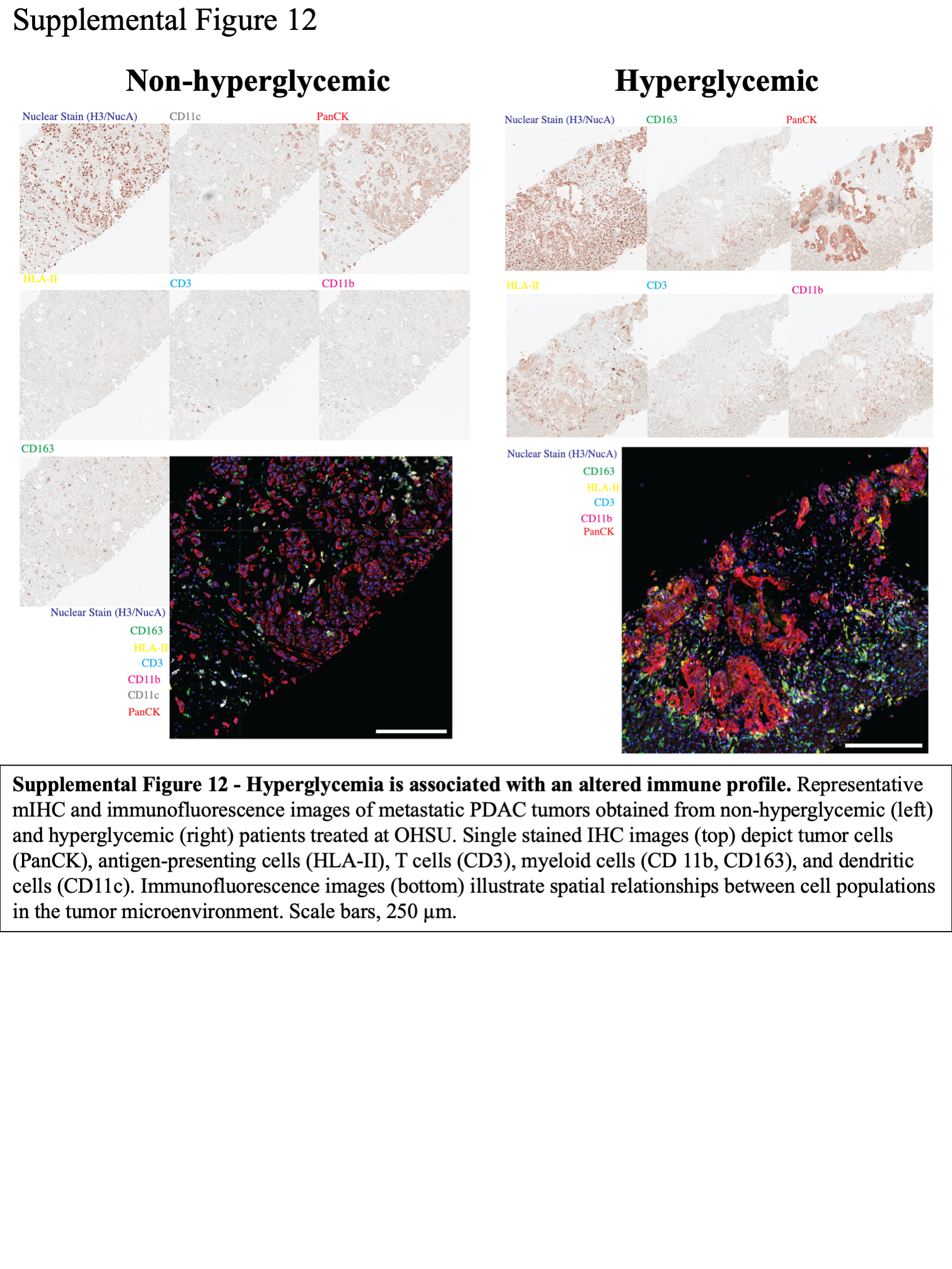

Supplement: Supplemental Figure 12 — Hyperglycemia is associated with an altered immune profile. Representative mIHC and immunofluorescence images of metastatic PDAC tumors obtained from non-hyperglycemic (left) and hyperglycemic (right) patients treated at OHSU. Single stained IHC images (top) depict tumor cells (PanCK), antigen-presenting cells (HLA-II), T cells (CD3), myeloid cells (CD 11b, CD163), and dendritic cells (CD11c). Immunofluorescence images (bottom) illustrate spatial relationships between cell populations in the tumor microenvironment. Scale bars, 250 µm. [file crc-25-0338_supplemental_figure_12_suppsf12.png]
